# In‐silico and in‐vitro evidence suggest LINC01405 as a sponge for miR‐29b and miR‐497‐5p, and a potential regulator of Wnt, PI3K, and TGFB signaling pathways in breast carcinoma

**DOI:** 10.1002/cnr2.1972

**Published:** 2024-01-15

**Authors:** Romina Norouzi, Zahra Mohamadzade, Rambod Norouzi, Radin Norouzi, Rezvan Esmaeili, Bahram M. Soltani

**Affiliations:** ^1^ Molecular Genetics Department, Faculty of Biological Sciences Tarbiat Modares University Tehran Iran; ^2^ Molecular Biosciences Department Autonomous University of Madrid Madrid Spain; ^3^ Biology Department Kharazmi University Tehran Iran; ^4^ Genetics Department, Center for Breast Cancer Research Motamed Cancer Institute Tehran Iran

**Keywords:** breast cancer, differential expression, LINC01405, sponge

## Abstract

**Background:**

Carcinoma of the breast, a prevailing factor in female mortality worldwide, involves dysregulation of lncRNAs and microRNAs.

**Aim:**

The main goal of this research was to predict and experimentally examine the LINC01405 expression status in breast cancer subtypes, along with investigation of its interaction with miR‐29b and miR‐497‐5p that results in regulating PI3‐Kinase, WNT, and TGF‐beta signaling pathways.

**Methods and Results:**

We performed a meta‐analysis of five GEO datasets, encompassing microarray and RNA‐seq data, to identify differentially expressed genes. The Cancer Genome Atlas transcriptome dataset was also analyzed to determine essential gene modules, associated with different stages of breast cancer by weighted gene co‐expression networks. In addition, networks of drug‐gene interactions were constructed to explore potential treatment options. LINC01405 as a microRNA sponge was chosen and examined. furthermore, downstream target genes were discovered.

Experimental validation consisted of plasmid constructs used in cell culture experiments, RT‐qPCR for expression analysis, and cell cycle assays. Our bioinformatics findings showed higher LINC01405 expression in Basal‐like triple‐negative breast carcinoma. In contrast, lower expression in Luminal samples was observed compared with normal samples, which was consistently observed in both breast cancer tissues and cell lines. LINC01405 expression level was correlated with miR‐29b and miR‐497 levels. The MDA‐MB‐231 cell line demonstrated higher LINC01405 expression and lower miR‐29b and miR‐497 expression levels. However, SKBR3 and MCF7 cells had lower LINC01405 expression and higher miR‐29b and miR‐497 levels, suggesting a regulatory role for LINC01405 as a competing endogenous RNA. This was experimentally confirmed when LINC01405 was overexpressed in SKBR3 cells, and the common target genes of miR‐29b and miR‐497 were upregulated. Additionally, LINC01405 upregulation led to the increased cell populations, proliferation, and upregulation of critical cancer‐related genes, including AKT1, AKT3, mTOR, WNT3A, SMAD3, CYCLIN D1, CYCLIN D2, BCL2, and GSK3B.

**Conclusion:**

We revealed the differential expression of LINC01405 in several types of breast cancer and its role in regulating signaling pathways, potentially via scavenging miRNAs. These findings clarified the role of LINC01405 in breast cancer development and identified potential therapeutic targets.

## 1 INTRODUCTION

Despite the invention of advanced and high‐tech technologies, breast cancer remains the leading cause of death in women worldwide. According to surface markers, researchers have separated breast cancer into four subgroups: HER2‐positive (HER2‐like), ER‐positive/HER2‐negative (corresponding to Luminal A and B), and Triple‐negative breast cancer, which is a type of breast cancer that does not have any of the receptors that are commonly found in breast cancer.^1^, ^2^, ^3^

Non‐coding RNAs have been the subject of several cancer studies with claims and proof of their remarkable influence on cancer. Recently, researchers have introduced several ncRNAs as biomarkers responsible for the onset, development, and progression of breast cancer.^4^, ^5^ Functional ncRNAs are categorized into four major groups: transfer RNAs, ribosomal RNAs, small RNAs such as microRNAs, siRNAs, snoRNAs, snRNAs, exRNAs, and long non‐coding RNAs.^6^

LncRNAs play vital roles in the physiologic or pathologic status of cells, such as proliferation, development, and immunity.^7^ They could adjust gene expression at the transcription, post‐transcription, and translation levels, as recent studies have implied their involvement in epigenetic regulation.^8^, ^9^ MicroRNAs are non‐coding, small RNAs with a length of 17 to 22 nucleotides that control the expression of genes. They, impede translational progress by degrading mRNA through Watson and Crick base pairing between 3'‐UTR MREs and miRNA seed sequences.^10^ According to their role in the cell, miRNAs are divided into two categories: “oncogenic microRNAs” which genetically promote tumor growth, and “tumor suppressive microRNAs” which are mainly in charge of suppressing oncogenes and overactivated signaling pathways.^11^, ^12^

DNA methylation is an important epigenetic process that involves the addition of a methyl group to cytosine at the C5 carbon residues (known as 5mC). Usually CpG sites within CpG islands in healthy cells are unmethylated. However, in cancer, there is often hypermethylation at these CpG sites, leading to the silencing of tumor suppressor genes.^13^

The exciting subject of sponging miRNA by lncRNAs has raised attention over the years, where lncRNAs appear as competing endogenous RNAs (ceRNAs) to scavenge miRNAs.^14^, ^15^ LINC01405, or loc100131138, on chromosome 12, is a 374‐bp intergenic lncRNA containing two exons and one intron. Researchers have documented the differential expression of LINC01405 in breast cancer through bioinformatics analysis. For instance, Yang et al.^16^ conducted next‐generation sequencing (NGS) on samples of her2‐enriched breast cancer. Their findings indicate that LINC01405 plays a pivotal role within the co‐expression network involving long non‐coding RNAs (lncRNA) and messenger RNAs (mRNA). In the same study, co‐expression networks were constructed between the tumor and normal groups based on Pearson correlation analysis. The K score was used to identify the central genes in this network, with higher scores indicating their regulatory roles within the network. The results demonstrated significant differences in the co‐expression network between tumor cells and tumor margin cells. Notably, LINC01405 exhibited the highest k‐core among the tumor groups, indicating its primary regulatory role in this gene network. Furthermore, experimental studies have explored its expression in other cancers, such as esophageal cancer.^17^ There is indeed a notable gap in research concerning the differential expression of LINC01405 in different subtypes of breast cancer and its regulatory roles. However, our study addresses this gap for the first time through a combined approach of bioinformatics and experimental analysis, providing valuable insights into both differential expression and methylation along with the regulatory functions of LINC01405 across various subtypes of breast cancer. This approach contributes to a deeper understanding of the role of LINC01405 in breast cancer and its potential implications for diagnosis and treatment.

Our research stands out in lncRNA studies because of its distinctive focus on identifying innovative targets within the lncRNA‐miR‐mRNA network and stage‐specific therapeutic drugs in breast cancer patients. Previous research has investigated LINC01405 in various cancer types^17^ but has not suggested specific drugs for targeting breast cancer at different stages. Using WGCNA and drug‐gene interaction analysis, our approach goes beyond conventional research. It seeks to identify targets specific to varying stages of breast cancer and the corresponding drugs tailored to these targets.

## 2 MATERIALS AND PROCEDURES

### 2.1 Bioinformatics analysis

#### 2.1.1 Data gathering and identification of differentially expressed genes using microarray and RNA‐seq (DEGs)

**FIGURE 1:**
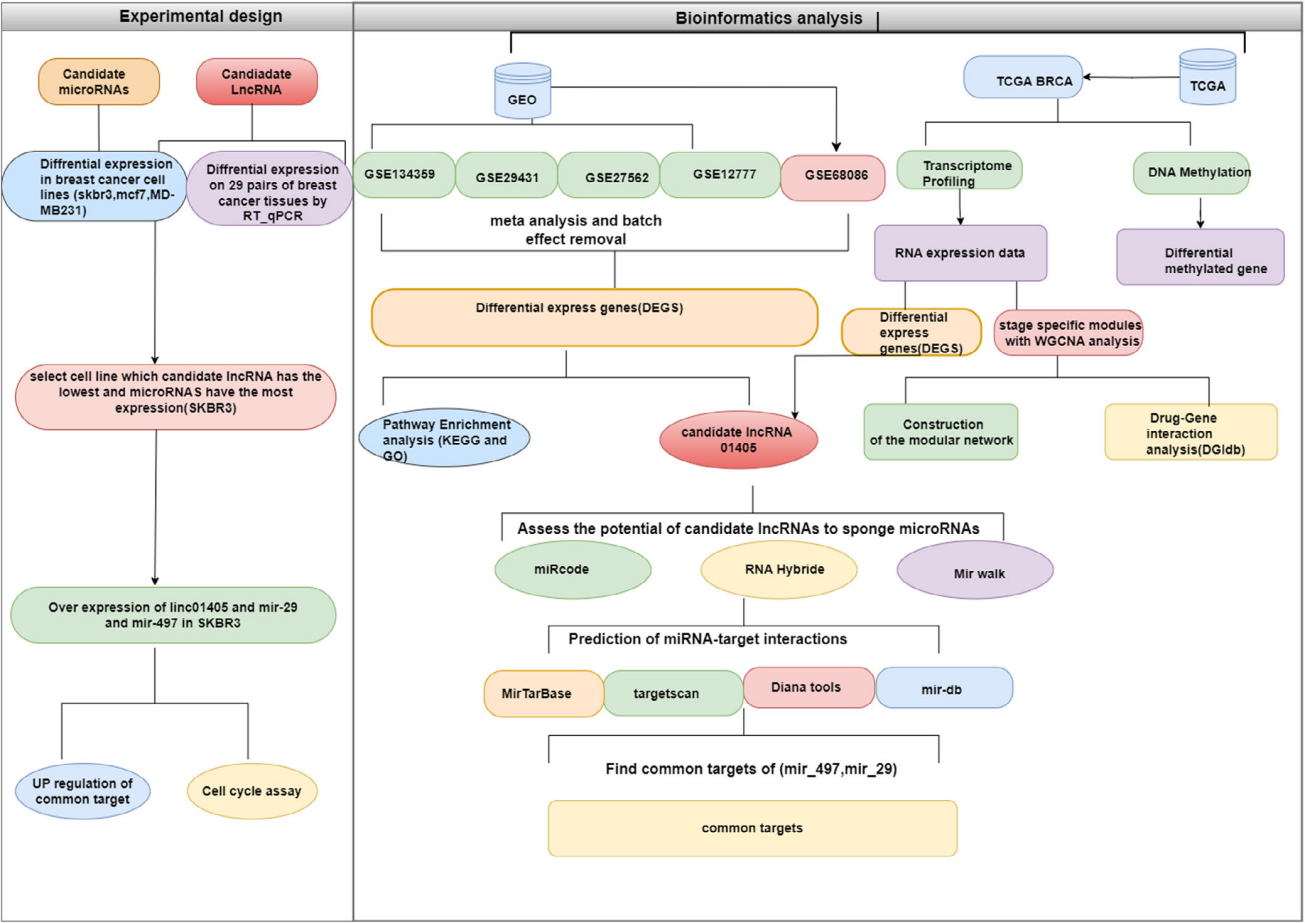
Flowchart of this study.

We designed bioinformatics to identify a functional lncRNA in breast cancer (Figure 1). We used five datasets (GSE134359, GSE29431, GSE27562, GSE12777, and GSE68086) from the GEO database. GSE134359 (Long noncoding RNA landscape in breast cancer, 75 adjuvant tumors, and 12 Adjacent normal tissue), GSE29431 (Identifying breast cancer biomarkers in breast cancer, 54 tumors, and 12 normal tissue), GSE27562 (Expression data from human PBMCs, 57 tumors, and 37 control peripheral blood mononuclear cells (PBMCs), and GSE12777 (Analysis of 51 human breast cancer cell lines gene expression profiles). Next, the selected microarray datasets were subjected to a meta‐analysis to identify DEGs.^3^, ^9^, ^18^ Before starting data processing, we annotated all genes and probe IDs as Entrez IDs to ensure consistency. In addition, we utilized the Sva package (version 3.40.0) in the R software to eliminate hidden batch effects and merge the four datasets. We used the Bioconductor‐based *R* package limma (version 3.48.3) to identify genes with differential expression from the microarray datasets. We applied an adjusted *P*‐value threshold of .05 and a log2 fold Change threshold of 1.41.

RNAseq data comprise one dataset, GSE68086 (Blood‐based, multiclass, and molecular pathway cancer diagnoses are made possible by RNA‐seq of tumor‐educated platelets), where breast cancer blood and control samples were candidates for further analysis. To avoid generating biased results between the two technologies (microarray and RNAseq), we preferred to analyze this platform separately from the microarray datasets. We decided not to pool the results and elucidated the mutual DEGs between the two platforms.

We performed two meta‐analyses of five datasets in the GEO database:

1. Between four microarray datasets.
2. Between the results (differential expression genes) of the meta‐analysis of microarray datasets and the RNAseq dataset (GSE68086).

#### 2.1.2 Functional enrichment analysis for meta‐analysis of microarray and RNAseq datasets

To identify the primary role and extract information about enriched pathways related to differentially expressed genes (DEGs), we utilized the GO database to evaluate the biological process (BP), cellular component (CC), and molecular function (MF) of each functional gene, aiming to comprehend their potential functions.^3^, ^9^, ^19^ To conduct this analysis, we used the ClusterProfler (V 4.0.2) *R* software package, which leverages an ontology for statistical analysis, and visualization of gene functional profiles. We used the Benjamini‐Hochberg approach and established the following standards: a *p*‐value of .05 and a *q*‐value under 0.05 for GO analysis of DEGs. Additionally, for GO pathway analysis, we considered a corrected *p*‐value of <.05 as the cutoff using the ClusterProfler.

### 2.2 Analysis of the TCGA dataset

The Cancer Genome Atlas (TCGA) is a public resource that displays cancer genetic changes in various cancer tissues. We accessed transcriptome data (Gene Expression Quantification) and clinical information of breast cancer patients (including 1229 samples, 1107 primary tumors, 114 normal solid tissue samples, and eight metastatic samples) from the TCGA database.^20^ R packages: TCGA biolinks, Summarized Experiment, and DT were used for data download and analysis. We conducted the normalization of RNAseq data using edgeR.

We performed expression data analysis for RNA between primary tumors and normal solid tissue using DESeq2. Only when the adjusted *p*‐value was less than .05, and the log‐fold change was >1.5, were differentially expressed genes (DEGs) reported.

### 2.3 Analysis of the weighted gene co‐expression network

A systems biology and bioinformatics method called weighted gene co‐expression network analysis (WGCNA) was used to create and examine co‐expression networks. WGCNA performs various tasks by examining the topology of modules associated with highly connected genes, including network creation, visualization, data simulation, and gene selection. Here, the BRCA transcriptome dataset was used as the input for WGCNA. We employed normalized count data to identify modules connected to stages I_IV, using Pearson's correlation matrices to build the adjacency matrix between all paired genes. A soft threshold power of 5 was used to generate a scale‐free adjacency matrix (scale‐free *R* ^2^ = 0.9), which also helped to reduce the correlation noise. Subsequently, a topological overlap matrix (TOM) was genereted from the adjacency matrix.

The modules with a correlation greater than 0.5 and a *p*‐value of .005 were retained, using the TOM‐based dissimilarity measure. A minimum size of 10 and a cut height of 0.9 were specified to allow for the combination of related modules. We were able to recognize key modules by using significantly differentially expressed genes (DEGs) with comparable expression patterns to cluster them into discrete gene modules using the average linkage hierarchical clustering approach. The association between module eigengenes (MEs), clinical factors (stages I to IV of breast cancer), and sample types were investigated in the search for clinically relevant modules.

### 2.4 Network of drug‐gene interactions

The Drug Gene Interaction Database (DGIdb42) was used to identify possible therapeutics for breast cancer. After conducting WGCNA analysis, a list of genes was selected in the consensus modules with colors (yellow, grey60, green) that contained DEGs within each module that met our criteria, and these genes were used as input for DGIdb. Subsequently, the network of gene‐drug interactions was extrated, and drug‐gene interaction networks were constructed and visualized using alluvial plotting.

### 2.5 Differential methylation analysis

Illumina Human Methylation 27 K array data from 313 primary breast cancer tumor solid tissues, 27 normal cases, and 27 342 methylation sites were used to analyze differential methylation. The beta value (*β*) was used as an index to quantify the methylation level, which identifies the ratio of the intensities of methylated and unmethylated alleles. Differential methylation analysis was performed between 27 normal and 313 tumor samples. Probes containing SNPs, chromosome X, and probes with more than 10% missing values were excluded from the analysis. The Wilcoxon rank‐sum test was used to determine the differentially methylated CpGs (DMCs), and the p‐values were adjusted using the FDR method. DMCs were reported if the mean methylation difference was >0.2 with an FDR of 5%.

#### 2.5.1 Integrative analysis of DNA methylation and gene expression

In this part of the study, Illumina Human Methylation 27 K methylation data and RNA‐seq gene expression data were integrated from the TCGA to detect breast cancer DNA methylation and expression markers. Different methylated CpGs (DMC) analysis was performed to understand the differentiation of DNA methylation in each group and their significance value. We performed a differential expression analysis (DEA) and detected the fold change in gene expression and their significance value. We integrated Differential expression and Differential methylation data, and set criteria in four sections. (Section 1) Hypo‐Down (logFC < (−1), delta beta < (−.2), FDR < (0.05), adjP.Val < (.05)), (section 2) Hypo‐Up (logFC < (1), delta beta < (−.2), FDR <0.05, adj pvalue<.05), section 3) Hyper‐Down ((logFC < (−1), delta beta > (.2), FDR <0.05, adjP.Val <.05)), section 3) Hyper‐Up (logFC < (1), delta beta > (.2), FDR <0.05, adjP.Val <.05).

#### 2.5.2 Assessment of candidate lncRNAs' potential to sponge microRNAs

The miRcode (http://www.miRcode.org/) and RNAhybrid (https://bibiserv.cebitec.uni-bielefeld.de/rnahybrid) databases were utilized to evaluate the potential of candidate lncRNA (LINC01405) as a microRNA sponge.

### 2.6 Prediction of miRNA‐target interactions

The miRNA target genes (miR‐497, miR29b) were identified using multiple databases, including Targetscan (https://www.targetscan.org/), mirTarBase (https://miRtarbase.cuhk.edu.cn/), miRdb (https://miRdb.org/), MiRwalk (http://miRwalk.umm.uni‐heidelberg.de/), and DIANA TOOLS (http://diana.imis.athena‐innovation.gr/). The shared set of genes targeted by both microRNAs was determined.

## 3 EXPERIMENTAL PROCEDURE

### 3.1 Plasmid constructs

The LINC01405 coding sequence was amplified by RT‐qPCR from cDNA derived from whole blood. The resulting insert was then inserted into the pTG19‐R/T vector and further subcloned into the pcDNA 3.1(+) vector using KpnI and XbaI restriction enzymes for cloning. Verification of all constructs was performed by sequencing. Additionally, the miR‐497 and miR‐29‐b sequences were cloned into the pEGFPC1 expression vector for experimental use.

### 3.2 Cell culture and transfection

SKBR3, a Her2‐enriched cell line obtained from the Institute Pasteur in Iran, was cultured in 12‐well plates at specified cell densities. Following this, the cells were transfected with an expression vector containing the LINC01405 sequence, using the TurboFectTM Transfection Reagent from Thermo. After a 4 h incubation period, the transfection medium was replaced with fresh media containing 10% FBS. The transfection efficiency was evaluated 24 h after transfection using a fluorescence microscope (Nikon Eclipse Te2000‐s).

### 3.3 Extraction of total RNA, synthesis of cDNA, and RT‐qPCR

Total RNA was extracted using RiboEx Total RNA Reagent, following the guidelines provided by the manufacturer (GeneAll Biotechnology). After treating 1 g of total RNA with DNase I, cDNA was synthesized to detect miRNAs. For this purpose, a polyA tail was added to the 3′ ends of RNAs using polyA polymerase (Takara, Japan), following the manufacturer's protocol. Subsequently, anchored oligo‐dt and random hexamer primers were introduced, and the mixture was incubated at 65 °C for 5 min. Reverse transcription was carried out at 42 °C for 70 min, followed by an incubation step at 72 °C for 10 min, and then held at 20 °C. For qPCR amplification, miRNA‐specific forward primers and a universal reverse primer recognizing the oligo‐dt anchor were added to the SYBR green master mix (BioFACT). During this process, no template controls were included, and all PCR reactions were performed in triplicate. The miRNA quantitative data were normalized to U48, whereas the mRNA quantification data were normalized to GAPDH. The fold change in gene expression was determined using the 2‐Ct method.

### 3.4 Cell cycle assay

SKBR3 cells were seeded in 12‐well plates and then transfected with pcDNA 3.1 (+) containing the LINC01405 fragment, alongside mock controls. After 48 h, the cells were detached using trypsin, and the collected cells were fixed in 70% ethanol and stored at 4 °C for 48 h. Subsequently, they were rinsed twice with cold PBS and exposed to PBS containing RNase A (10 mg/mL) for 1 h at 37 °C. Following this, the cells were stained with propidium iodide (40 mg/mL). The cell proliferation index was measured using flow cytometry, and flow cytometry software was used to analyze and calculate the proliferation index (Figure 1).

### 3.5 Statistical evaluation

The data are stated as the average ± standard error (SE). Student‐paired *t*‐tests or independent t‐tests were used to compare the measured data between groups. Software from IBM, Armonk, New York, in version 22 of SPSS and *R* was used for all statistical studies. Using GraphPad Prism 5.0 (GraphPad, La Jolla, CA), the findings were visually shown. The Wilcoxon test in R software generated the scatter plot and paired plot. A *p*‐value of less than .05 was considered statistically significant for all two‐tailed tests.

## 4 RESULTS

### 4.1 Meta‐analysis

Initially, the log2 raw expression data obtained from breast cancer samples were imported into R software. Subsequently, the data were normalized using the Normalize‐Quantiles function.

**FIGURE 2:**
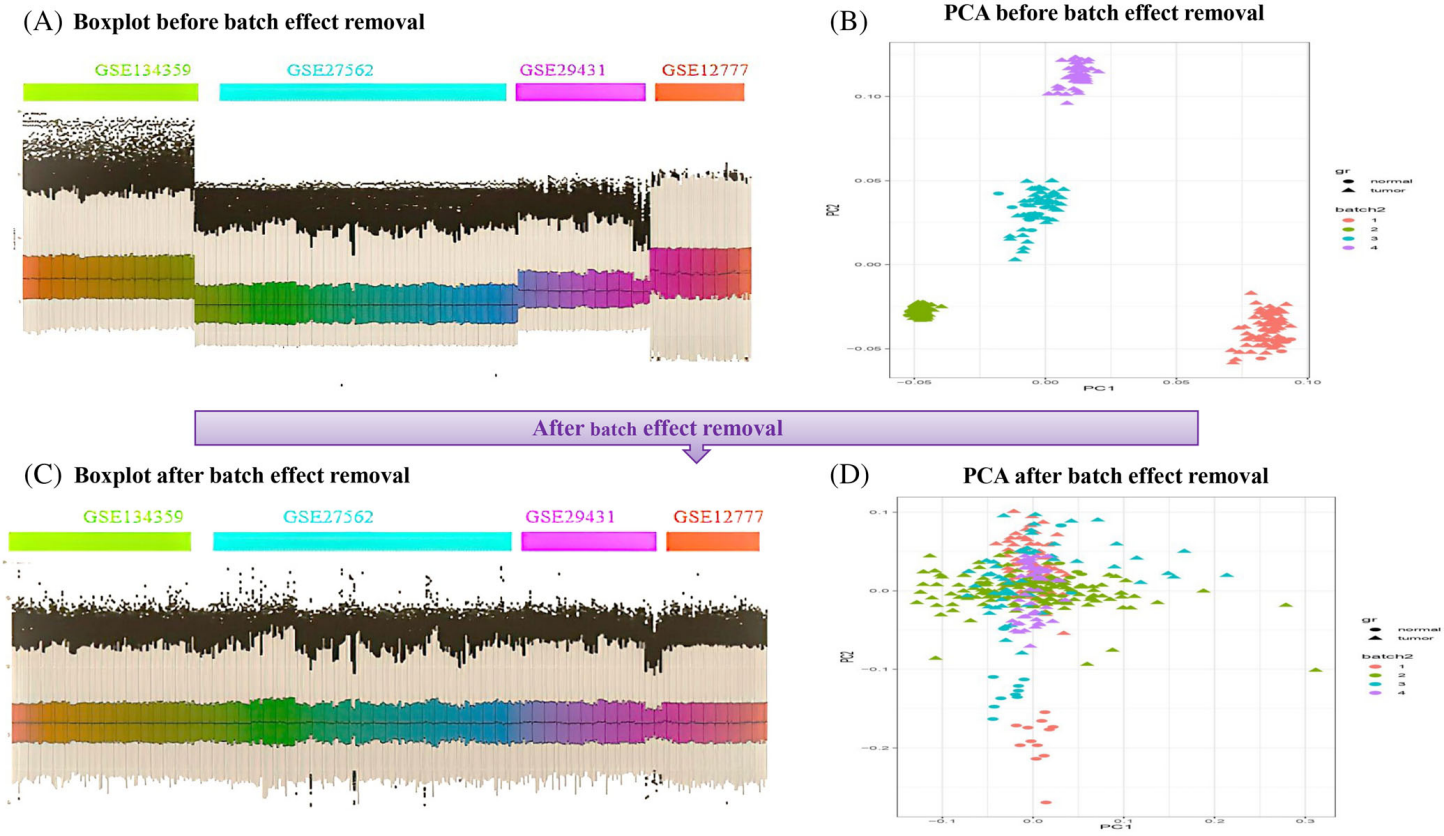
Microarray meta‐analysis quality control, Box plots, and PCA of data before and after batch effect correction. (A) Shows the boxplot of four microarray datasets before batch effect removal. (B) Shows the PCA analysis of four microarray datasets before batch effect removal. (C) Presents boxplot of four microarray datasets after batch effect removal. (D) Presents PCA analysis of four microarray datasets after batch effect removal.

Next, the four microarray datasets were combined, and the widely used Combat function available in the *R* (SVA) package was employed to remove any underlying batch effects. As a result, a box plot representing 366 samples before and after batch effect removal was produced (Figure 2A,C).

Because the data had a high dimensionality, which hardened the identification of breast cancer tumor samples from their normal counterparts, principal component analysis (PCA) was utilized for unsupervised classification following the normalization of the four datasets. The process helped us to discriminate breast tumors and normal tissue samples from each other before and after batch effect removal. unsupervised classification through PCA revealed successful discrimination between tumor tissues of each cancer type and normal samples. Figure 2B,D).

**FIGURE 3:**
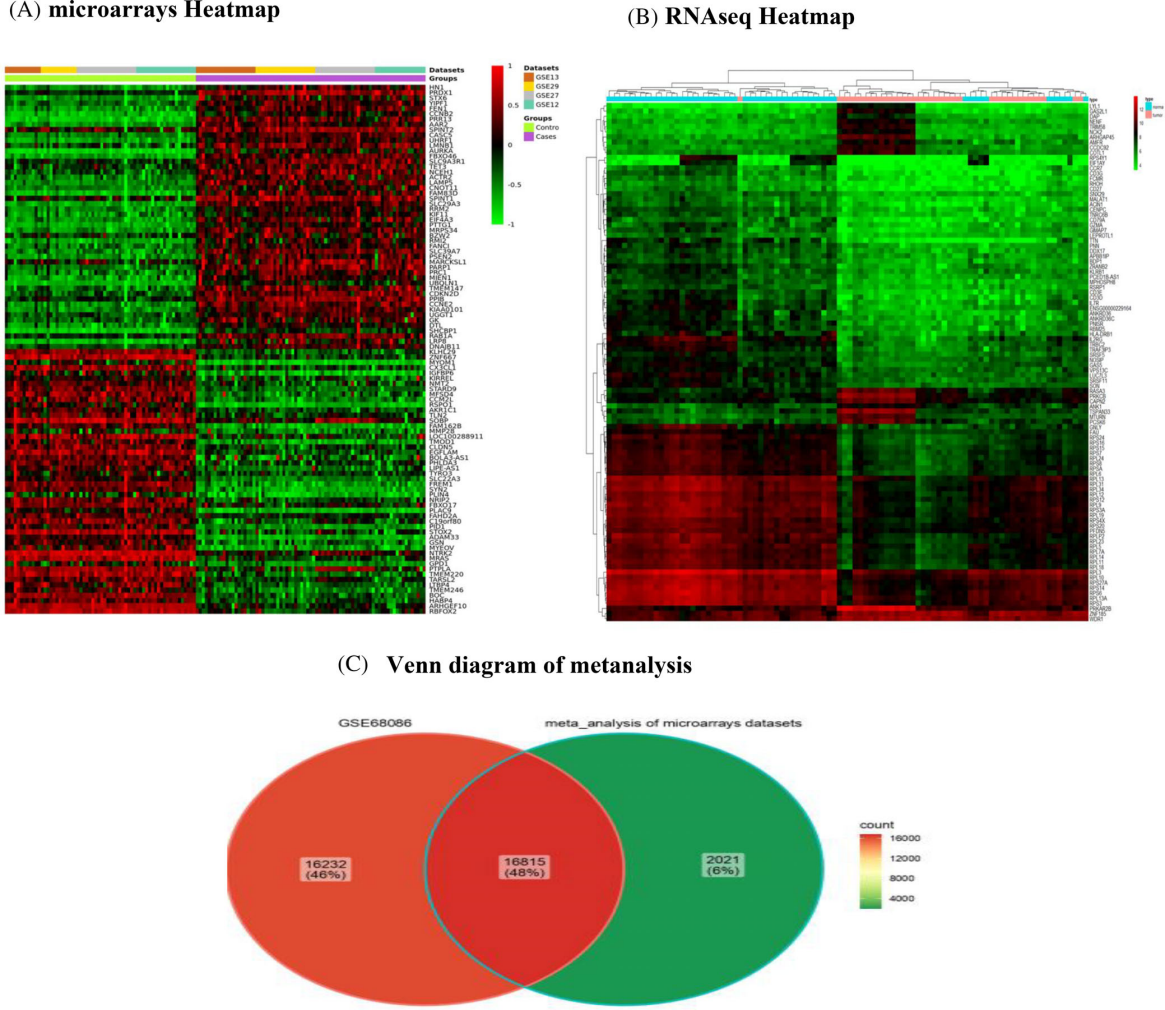
Heat map representing and Venn diagram of differentially expressed genes in breast cancer. (A) Meta‐analysis of individual microarray data sets indicated that 18 837 genes were differentially expressed in breast cancer. (B) Comparative individual RNAseq data analysis from GEO(GSE68086) indicated that 10 227 genes were differentially expressed in breast cancer. (C) Venn diagram of intersection between DEGs in the microarray meta‐analysis of an RNAseq (GSE68086) dataset, there are 16 815 (48%) genes shared in both datasets. Also, 16 232 DEGs are GSE68086‐specific, while 2021 genes are microarray‐specific.

In this section, the expression patterns of differentially expressed genes (DEGs) across various datasets and conditions were displayed as a heatmap using the Complex Heatmap package in R. For the meta‐analysis of each dataset, we used a heatmap to visually represent the correlation in expression patterns for a specific subset of genes in the microarray datasets (Figure 3A). Also, we draw the expression profile heat map for the DEGs in GSE68086 (Figure 3B) and the expression profile heatmap for the DEGs in TCGA datasets (Sup Figure S1). In between, our results emphasized the downregulation of LINC01405 in breast cancer (Sup Tables S1–S3).

We revealed that genes such as LOC10028891, LIPE‐AS1, and BOLA3‐AS1 also exhibit differential expression between breast carcinoma and adjacent normal tissue samples (Figure 3A). Furthermore, the GNLY, PNN, and PCED1B‐AS1 genes were among the critical DEGs in GSE68086 (Figure 3B). These DEGs in microarray datasets (Sup Figure S2A), RNAseq data (Sup Figure S2B), and TCGA data are visualized in volcano plots (Sup Figure S2C).

The Venn diagram of the intersection between the meta‐analysis of the four microarray datasets and the RNAseq (GSE68086) dataset showed that 16 815 (48%) genes were mutually expressed between the two meta‐analytic studies (Figure 3C).

### 4.2 GO and KEGG pathway enrichment analysis

**FIGURE 4:**
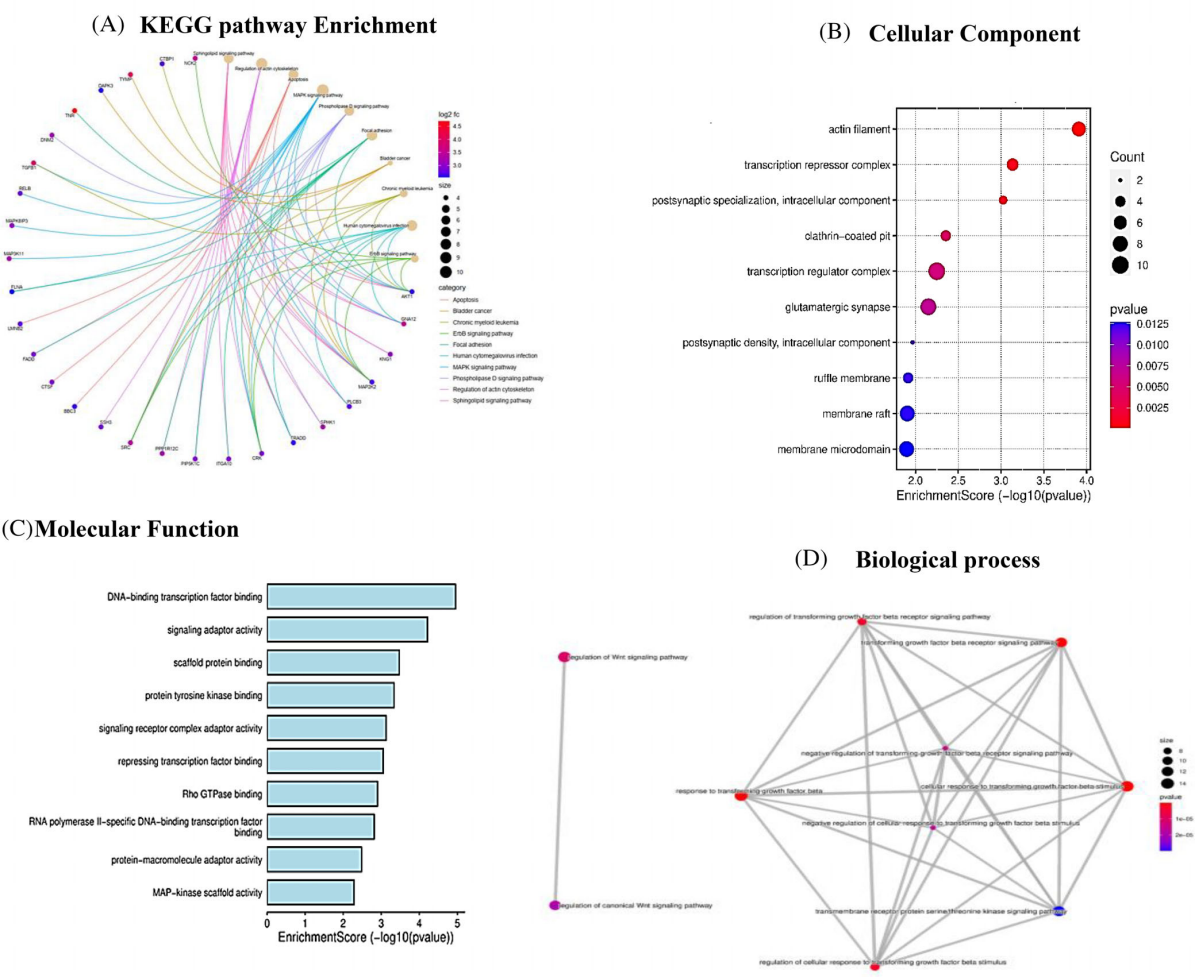
Pathway enrichment plot for the intersected DEGs of breast cancer. (A) Cnetplot KEGG pathway enrichment produced from the intersected DEGs (microarray meta‐analysis and RNAseq (GSE68086). Accordingly, MAPK, Focal adhesion, apoptosis, sphingolipid, and ERbB signaling are the most prominent pathways. (B) Dot plot shows an overview of the cellular components of GO analysis. Accordingly, actin filament and transcription repressor complex systems are the most prominent cellular components involved in breast cancer incidence. (C) Bar plot shows an overview of the molecular function of GO analysis. Accordingly, DNA‐ binding transcription factors are the most important molecular functions in breast cancer incidence.(D) According to GO analysis, TGFB, Wnt, and serine/threonine signaling are in the most important biological processes involved in breast cancer.

To categorize the function of DEGs in cellular pathways, we illustrated a Cnetplot that depicts the linkages of genes and biological concepts (KEGG pathways) as a network. Based on the log2 fold change of the genes, the ERbB signaling pathway was introduced. Accordingly, NCK2, AKT1, CK, and SRC were the four essential genes with differential expression associated with the ERbB signaling pathway (Figure 4A). The study employed gene ontology (GO) analysis, which involves biological processes (BP), cellular components (CC), and molecular functions (MF) are the three categories. The purpose of this study was to gain deeper insights into the roles of 16 815 genes that showed differential expression (DEG) (refer to Figure 4B (CC), 4C (MF), and 4D (BP)).

For this dataset, 2,88 biological processes, 286 cellular components, and 393 molecular functions were enriched. The significance level was set at a *p*‐value of less than .05.

#### 4.2.1 Candidate LncRNA

Based on the data generated from the meta‐analysis and TCGA, several lncRNAs that showed remarkable expression differences within the breast cancer malignant and normal tissues were chosen for further analysis. LncRNA disease analysis indicated that ectopic expression of LINC01405 is associated with several cancers, such as cervical cancer, lung cancer, and hepatocellular carcinoma. However, it is most strongly associated with breast cancer (Sup Figure S3).

### 4.3 WGCNA identifies necessary modules relevant to stages I–IV

**FIGURE 5:**
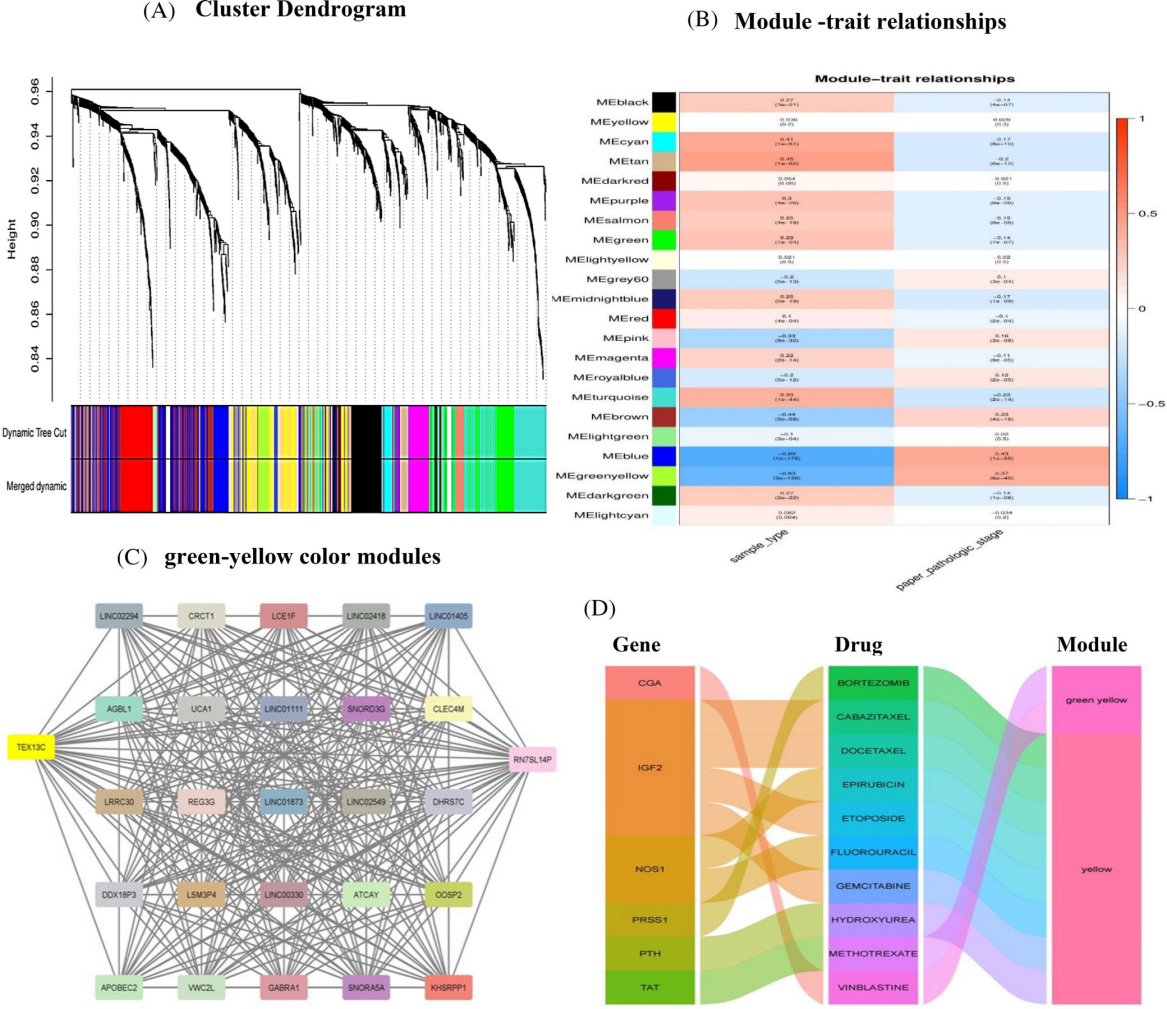
WGCNA analysis PLOT based on the tumor stages of breast cancer. (A) Clustering dendrograms of robust DEGs and related modules based on a dissimilarity measure (1‐TOM). (B) Heatmap of the correlation between module eigengenes and stages I to IV (as clinical traits) in BRCA. Data shows that, unlike other modules, MEbrown and MElightgreen modules do not correlate with cancer stages. (C) Representation of green‐yellow color modules produced by WGCNA analysis in breast cancer. (D) Data shows only green and yellow modules interact with current therapy drugs.

First, outlier samples that lacked biological significance were found and eliminated using a sample clustering tree. Then, utilizing a soft‐thresholding power of *β* = 5 (with a scale‐free *R* ^2^ of 0.9) and a cut height of 0.4, 22 modules, each represented by a different color, were discovered, along with DEGs that did not cluster subsequently. The, yellow (162 DEGs), gray 60 (14 DEGs), and green‐yellow (36 DEGs) modules exhibited significant associations with clinical information based on stages I to IV, as determined through module‐trait relationship evaluation (Figure 5A,B,C and Sup Figures S4–S7).

### 4.4 Construction of the modular network

Having identified the crucial modules, namely, yellow (162 DEGs), gray (14 DEGs), and green‐yellow (36 DEGs), a network was constructed to visualize the most significant candidate modules associated with breast cancer. In this network, nodes represent differentially expressed genes. Three separate networks were presented: a green‐yellow network, (Figure 5C), a gray network (Sup Figure S6), and a yellow network (Sup Figure S7). The gene LINC01405 was found within the green‐yellow module (Figure 5C).

### 4.5 Detection of potential drug‐target networks

Following the determination of modules in breast cancer, possible drug‐target interactions were investigated. Figure 5D depicts prospective drug‐target networks built using particular DEGs from the DGIdb database. The medications in the plot are chemotherapy treatments used to treat various malignancies, particularly gemcitabine, which is utilized in combination therapy for breast cancer.

### 4.6 Analysis of the relationship between DNA methylation and gene expression through integration

Analysis of DNA methylation and gene expression data revealed 875 genes exhibiting differential methylation and expression based on predefined criteria. Among these genes, 345 genes were found to be hypermethylated and downregulated, whereas 336 genes displayed hypermethylation and upregulation. Additionally, 147 genes exhibited hypomethylation and downregulation, and 49 genes showed hypomethylation and upregulation (Sup Table S5). These findings provide insights into the complex regulatory mechanisms involving DNA methylation and gene expression in the studied context. In addition, we demonstrated integration data in a scatter plot of mean methylation difference versus log2 expression (Supplementary Figure S8A). In the last step, we observed that LINC01405 showed hypomethylation status (median = 0.68) in the case group, whereas it was hypermethylated in normal control (median = 0.62) (Supplementary Figure S8B).

### 4.7 Confirmation of bioinformatics analysis using experimental results

**FIGURE 6:**
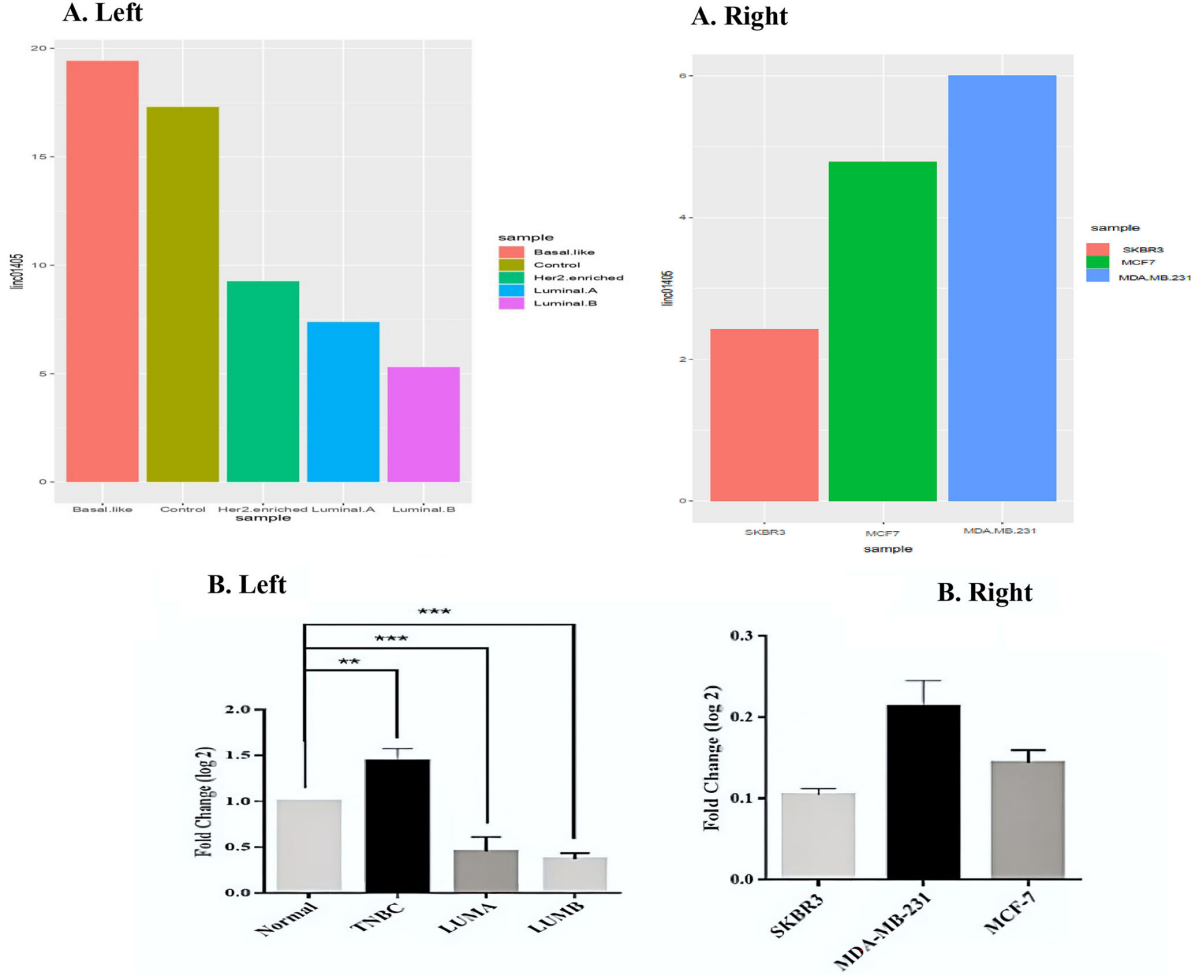
Expression analysis of Linc01405 in breast cancer tissue and cell line samples. (A) (Left) Expression of linc01405 in GSE134359 in the different subtypes of breast cancer and control, adopted from the microarray and RNAseq data. (Right) The expression of linc01405 in three breast cancer cell lines (GSE12777). Accordingly, LINC01405 is highly expressed in basal‐like cells while downregulated in Luminal B cancer types and related cell lines. (B) (Left) RT‐qPCR experimental validation of LINC01405 expression in 29 breast cancer tissue samples and three (SKBR3, MD‐MB231, and MCF7) cell lines (Right). Consistently, LINC01405 is highly expressed in basal‐like (TNBC) while being downregulated in Luminal B cancer types and related cell lines.

In subtype categorization, GSE134359 (Long noncoding RNA landscape in breast cancer, 75 adjuvant tumors, and 12 Adjacent normal tissues) dataset analysis indicated that the expression level of LINC01405 in Triple‐negative breast cancer (Basal‐like) tissues was higher than that in the control. However, its expression in the Her2‐enriched and Luminal tissues subclasses was lower than that the control, whereas its expression level in the Her2‐enriched tissues subtype was higher than that in the Luminal tissues subtype (Figure 6A left). This pattern was experimentally validated in tissue samples to a certain extent, where we confirmed the highest expression level of LINC01405 in TNBC compared with Luminal A and B subtypes (Figure 6B left). We also analyzed LINC01405 expression levels in cell lines of breast cancer. Our bioinformatics analysis revealed that the LINC01405 expression level in cell lines of breast cancer to some extent follows the breast cancer tissues subtype pattern, where the highest expression of this lncRNA was observed in MDA‐MB‐231(Triple‐negative cell line) compared to the Her2‐enriched and the Luminal A cell line, (SKBR3) and (MCF7) (Figure 6A right). This also showed consistency with experimental data, in which LINC01405 had the highest expression level in the cell line of MDA‐MB‐231 compared with SKBR3 and MCF7 cell lines (Figure 6B right).

**FIGURE 7:**
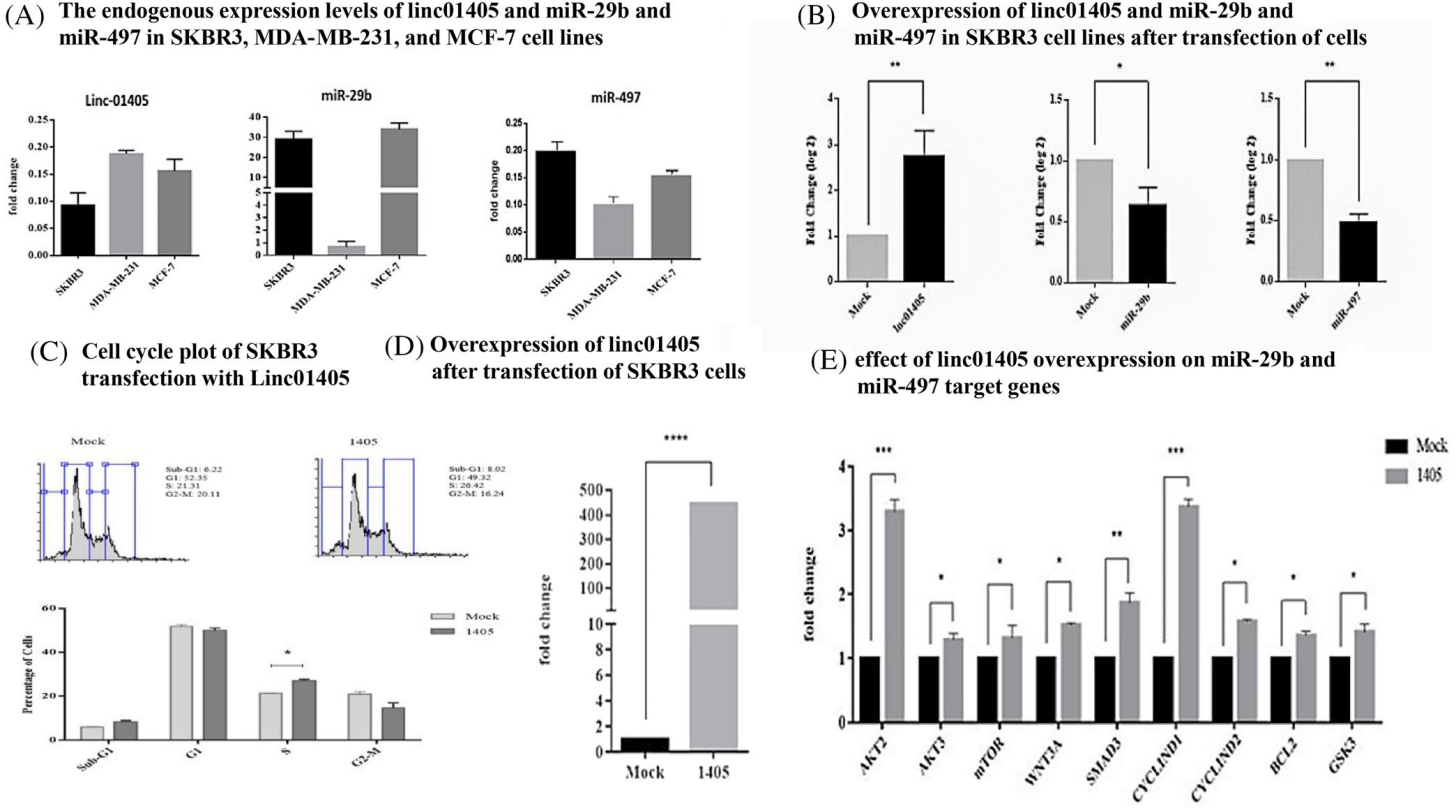
Endogenous and overexpressed Linc01405 and miR‐29b and miR‐497 and their effects on genes targeted by microRNAs after transfection in the SKBR3 cell line. (A) The endogenous expression levels of linc01405 and miR‐29b and miR‐497 in SKBR3. MDA‐ MB‐231, and MCF‐7 cell lines. (B) overexpression of linc01405 and miR‐29b and miR‐497 in SKBR3 cell lines after transfection of cells. (C) Cell cycle plot of SKBR3 after transfection with Linc01405. (D) Overexpression of linc01405 after transfection of SKBR3 cells. (E) The effect of linc01405 overexpression on miR‐29b and miR‐497 target genes.

Our experimental observation showed that the highest expression of LINC01405 in the cell line of MDA‐MB‐231 came with lower miR‐29b and miR‐497 expression levels. Conversely, lower levels of LINC01405 expression were observed in SKBR3 and MCF7 cell lines, with higher miR‐29b and miR‐497 expression levels (Figure 7A).

To confirm the ceRNA effect of LINC01405, we overexpressed LINC01405 within SKBR3 cells and observed a significant drop in miR‐29b and miR‐497 expression levels (Figure 7B) as well as an increased expression level of these miRNAs common target genes (Figure 7E).

Transient manual upregulation of LINC01405 in SKBR3 cells led to increased cell populations and induced cell proliferation (Figure 7C). This oncogenic potential of LINC01405 was further validated when we reported significant upregulation of AKT1, AKT3, mTOR, WNT3A, SMAD3, CYCLIN D1, CYCLIN D2, BCL2, and GSK3B in these cells (Figure 7D, E). It is worth mentioning that LINC01405 may serve as a biomarker for breast cancer based on An ROC curve analysis (Sup Figure S9).

Using the miRcode database, we found that LINC01405 contains one highly conserved binding sequence for miR‐29b and three binding sites (with an average conservation score) for miR497 (Sup Figure S10A). Additionally, we used the RNA hybrid database to predict the base pairing between LINC01405 and miR‐29b and miR‐497(Sup Figure S10B). Common genes targeted by both microRNAs (miR‐29b and miR_407), include, AKT1, AKT3, mTOR, WNT3A, SMAD3, CYCLIN D1, CYCLIN D2, BCL2, and GSK3B (Sup Figure S10C). In supplemental Table S4, a list of the primers utilized in this investigation is provided.

## 5 DISCUSSION

Breast cancer is a multifaceted disease that requires further understanding of its underlying mechanisms. Current endeavors have been concentrated on exploring the molecular aspects participating in the onset and advancement of breast cancer.^3^, ^9^, ^21^ Hundreds of molecular elements, such as proteins and RNAs, are involved in disease generation.^22^ In this study, we conducted a meta‐analysis on four microarrays and one RNAseq dataset from GEO, and an analysis of RNAseq data from TCGA separately. We found a list of differentially expressed genes and non‐coding RNAs from each of them and selected LNC01405 among them to validate its function in breast cancer. LINC01405 was already known as a down‐regulated lncRNA in esophageal carcinoma.^17^ Furthermore, it was previously introduced as a prognostic marker in tongue carcinoma.^23^ Based on the meta‐analysis results, we understood that LNC01405 showed the highest level of expression in cases of Triple‐negative breast cancer samples. We were able to validate the experimental phase of the research by successfully confirming it in 29 pairs of breast tissue and adjacent normal tissue samples. It is evident that LINC01405 functions in a network, and by drawing this network, it was suggested that the co‐expression network between the group's tumor and tumor margin had tremendous differences. Previously, it was reported that LINC01405 has the highest score (k‐core) among the tumor groups, which shows the prominent regulatory role of LINC01405 in breast cancer. Additionally, our bioinformatics analysis suggested that LINC01405 is a sponge for miR‐29b and miR‐497. Wang et al., demonstrated that miR‐29b is upregulated in breast cancer cells to induce migration.^24^ Also, it was reported to be a tumor suppressor, which showed a negative correlation with DNMT3A.^25^ Liu et al. suggested that the interaction between CAF1 and miR‐29b to induces proliferation in metastasis.^26^ On the other hand, miR‐497 could directly target Bcl‐w and induce apoptosis,^27^ whereas more tumor suppression effects were reported for miR‐497 by targeting HIF‐1α and preventing angiogenesis in breast cancer cells.^28^

Our claim was first predicted by MiRcode, RNA hybrid, and MiR walk software and confirmed when the overexpression of LINC01405 caused the expression of miR‐29b and miR‐497 to decrease significantly. Further evidence of LINC01405's ability to act as a scavenger was provided when we observed a significant rise in the expression of essential target genes for both miRNAs. MiR‐29b has been demonstrated to suppress tumor growth by targeting the AKT2 isoform.^29^ Our study found upregulation of AKT1 and AKT3, which are involved in the signaling pathway of PI3K, and previous studies have demonstrated overactivation of the PI3K pathway in breast cancer. The enhanced mTOR expression, induced by miR‐29b and miR‐497, may result from AKT overexpression and phosphorylation. Activation of the PI3K/AKT/mTOR pathway in breast cancer has been reported to induce cell proliferation, and in this research, the increased proliferation might result from the induced PI3K/AKT/mTOR pathway. The enhanced mTOR expression, induced by miR‐29b and miR‐497, may be the result of AKT overexpression and phosphorylation.^30^ miR‐497 is also reported to be a negative regulator of the TGFB signaling pathway. This pathway is activated in breast cancer, and when negative regulators such as miR‐497 are diminished by the aid of an lncRNA scavenger, hyperactivation of the pathway is expected.^31^ The increased expression of CCND1 and CCND2, along with AKT1, AKT3, and mTOR, may trigger cell proliferation, as observed by flow cytometry assay.^32^

The canonical Wnt signaling pathway is induced by Wnt1, Wnt2, Wnt3 and Wnt3a.^33^ Following the upregulation of Linc‐01405, we reported the upregulation of Wnt3a and GSK‐3 as significant proteins involved in Wnt signaling. Accordingly, we suggest the oncogenic role of LINC01405 in activating the key participants in the Wnt signaling pathway and the consequent proliferation of cancer cells. Bcl2 is known to suppress cancer cell apoptosis, and our further evidence for the oncogenic role of LINC01405 comes from Bcl2 upregulation following LINC01405 overexpression in the cell lines of SKBR3. Cellularly, overexpression of LINC01405 resulted in an increased *S*‐phase cell population, consistent with the Bcl2 upregulation effect.^34^

Taken together, we hypothesized that LINC01405 could play a significant role in breast cancer. Via dataset analysis, we predicted a cancer promoter role for LINC01405. Our experimental evidence showed that LINC01405 promoted cancer cell proliferation, suggesting an oncogenic effect for this lncRNA. However, when we consider LINC01405 as a player of a regulatory network where it might regulate miR‐29b and miR‐497 (which are reported both as tumor suppressors and oncogene in several breast cancer studies), it is not logical to emphasize a strict effect (tumor suppressor or oncogene effect) for LINC01405. Here, we suggested that LINC01405 sponges miR‐29b and miR‐497 through which, it upregulated common target genes of these miRNAs, resulting in the upregulating of Wnt, PI3K, and TGFB signaling. Here, we took the first step to investigate the effect of LINC01405 on breast cancer, but it is necessary to conduct comprehensive study programs to find its role in different cancers and more breast cancer samples.^35^, ^36^, ^37^, ^38^

**FIGURE 8:**
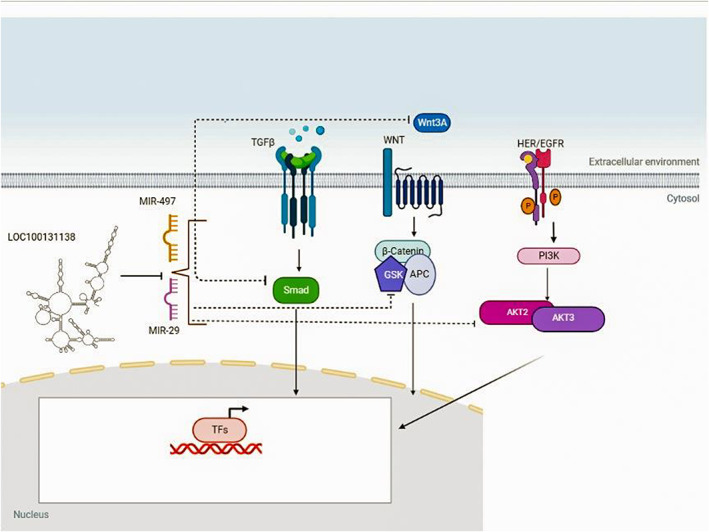
Schematic view of linc01405 in the cell.

Details associated with the LINC01405 (Loc100131138) function in the cell are represented by a schematic (Figure 8).

## AUTHOR CONTRIBUTIONS

**Romina Norouzi:** Formal analysis (equal); investigation (equal); resources (equal); software (equal); validation (equal); visualization (equal); writing – original draft (equal); writing – review (equal). **Zahra Mohamadzade:** Formal analysis (equal); investigation (equal); resources (equal); software (equal); validation (equal); visualization (equal); writing – original draft (equal). **Rambod Norouzi:** Formal analysis (equal); investigation (equal); resources (equal); software (equal); validation (equal); visualization (equal); **Radin Norouzi:** Formal analysis (equal); investigation (equal); resources (equal); software (equal); validation (equal); visualization (equal); **Rezvan Esmaeili:** Advising the research. **Bahram M. Soltani:** Conceptualization (lead); investigation (equal); project administration (lead); supervision (lead); writing – review and editing (equal).

## FUNDING INFORMATION

This reserach was partially supported by Tarbiat Modares University financial aids.

## CONFLICT OF INTEREST STATEMENT

The authors declare that they have no financial conflicts of interest with any organization related to the content discussed in the manuscript.

## ETHICS STATEMENT

The research involving human subjects received approval from the Motamed Cancer Institute (MCI) in Tehran, Iran.

## PARTICIPANT AGREEMENT

All participants/patients provided written informed consent to participate in this study.

## Supporting information


**Supplementary Figure 1.** Heatmap for Comparative individual RNAseq data analysis from the TCGA database indicated 17 364 genes were differentially expressed in breast cancer.
**Supplementary Figure 2.** Volcano plot representing DEGs in breast cancer datasets
Represents Volcano plot of DEGs based on four microarray datasets(A), for DEGs in the RNAseq (B), and dataset for DEGs in TCGA (C).
**Supplementary Figure 3.** linc01405 disease relation plot: linc01405 disease assessment based on LncRNADisease v2.0 (http://www.rnanut.net/lncrnadisease/index.php/hom).
**Supplementary Figure 4.** Scatter plot of the key modules (yellow, gray60, green‐yellow) representing breast cancer stages.
**Supplementary Figure 5.** Shows the clustering of module eigengenes, in which 22 modules are produced. Only two modules (MEbrown and MElightgreen) are lower than the cutoff (red line).
**Supplementary Figure 6.** Representation of gray modules produced by WGCNA analysis in breast cancer.
**Supplementary Figure 7.** Representation of yellow modules produced by WGCNA analysis in breast cancer.
**Supplementary Figure 8.** (A) Scatter plot of mean methylation difference versus log2 expression. (B) Methylation status of Linc01405.
**Supplementary Figure 9.** ROC curve analysis of Linc01405 based on Real‐Time data, with the area under the curve at 70%.
**Supplementary Figure 10.** Bioinformatics analysis of microRNAS sponges and targets. (A) MiRcode results from interactions between linc01405 and miR‐29b and miR‐497. (B) Base pairing prediction between linc01405 and miR‐29b and miR‐497. (C) Common mircroRNAS (mir‐497, mir‐29b target genes result.

**Supplementary Table 1.** The top 20 most strongly up‐ or down‐regulated DEGs by meta‐analysis of microarray data.
**Supplementary Table 2.** The top 20 most strongly up‐ or downregulated DEGs in a meta‐analysis of microarray data and GSE68086.
**Supplementary Table 3.** The top 20 most strongly up‐ or down‐regulated DEGs in TCGA
**Supplementary Table 4.** list of primers.
**Supplementary Table 5.** differentially expressed and methylated genes.

## Data Availability

This published article contains all the data that was generated or analyzed during the course of this study.
