# Supplementary material for: In‐silico and in‐vitro evidence suggest LINC01405 as a sponge for miR‐29b and miR‐497‐5p, and a potential regulator of Wnt, PI3K, and TGFB signaling pathways in breast carcinoma
Source: Cancer Rep (Hoboken). 2024 Jan 15;7(2):e1972. doi: 10.1002/cnr2.1972 (PMC10849987; doi:10.1002/cnr2.1972)
Supplement: Supplementary file 1 — Supplementary Figure 1. Heatmap for Comparative individual RNAseq data analysis from the TCGA database indicated 17 364 genes were differentially expressed in breast cancer. Supplementary Figure 2. Volcano plot representing DEGs in breast cancer datasets Represents Volcano plot of DEGs based on four microarray datasets(A), for DEGs in the RNAseq (B), and dataset for DEGs in TCGA (C). Supplementary Figure 3. linc01405 disease relation plot: linc01405 disease assessment based on LncRNADisease v2.0 (http://www.rnanut.net/lncrnadisease/index.php/hom). Supplementary Figure 4. Scatter plot of the key modules (yellow, gray60, green‐yellow) representing breast cancer stages. Supplementary Figure 5. Shows the clustering of module eigengenes, in which 22 modules are produced. Only two modules (MEbrown and MElightgreen) are lower than the cutoff (red line). Supplementary Figure 6. Representation of gray modules produced by WGCNA analysis in breast cancer. Supplementary Figure 7. Representation of yellow modules produced by WGCNA analysis in breast cancer. Supplementary Figure 8. (A) Scatter plot of mean methylation difference versus log2 expression. (B) Methylation status of Linc01405. Supplementary Figure 9. ROC curve analysis of Linc01405 based on Real‐Time data, with the area under the curve at 70%. Supplementary Figure 10. Bioinformatics analysis of microRNAS sponges and targets. (A) MiRcode results from interactions between linc01405 and miR‐29b and miR‐497. (B) Base pairing prediction between linc01405 and miR‐29b and miR‐497. (C) Common mircroRNAS (mir‐497, mir‐29b target genes result. [file CNR2-7-e1972-s002.docx]

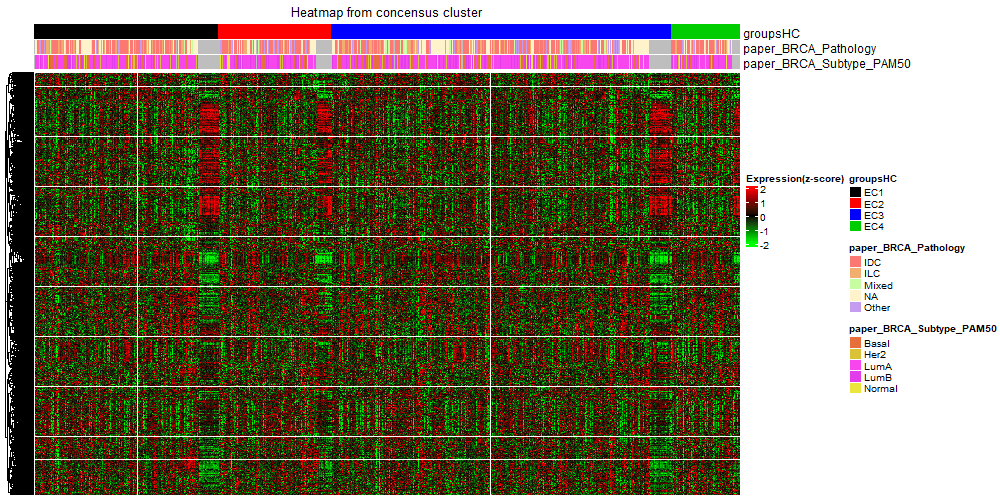


**Supplementary Fig.1) Heatmap for Comparative individual RNAseq data analysis from the TCGA database indicated 17364 genes were differentially expressed in breast cancer**


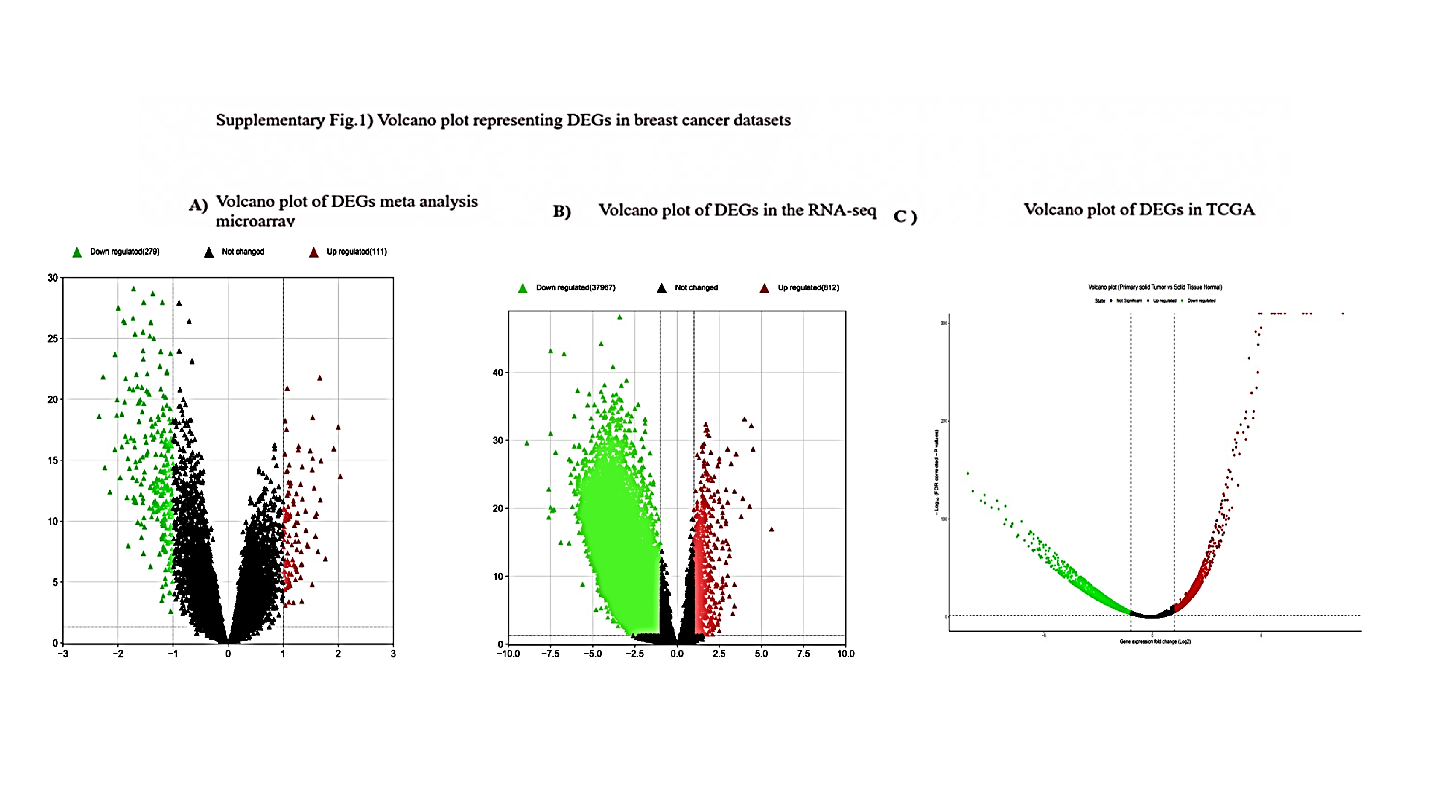


**Supplementary Fig.2) Volcano plot representing DEGs in breast cancer datasets**

Represents Volcano plot of DEGs based on four microarray datasets(A), for DEGs in the RNAseq (B), and dataset for DEGs in TCGA (C).

**
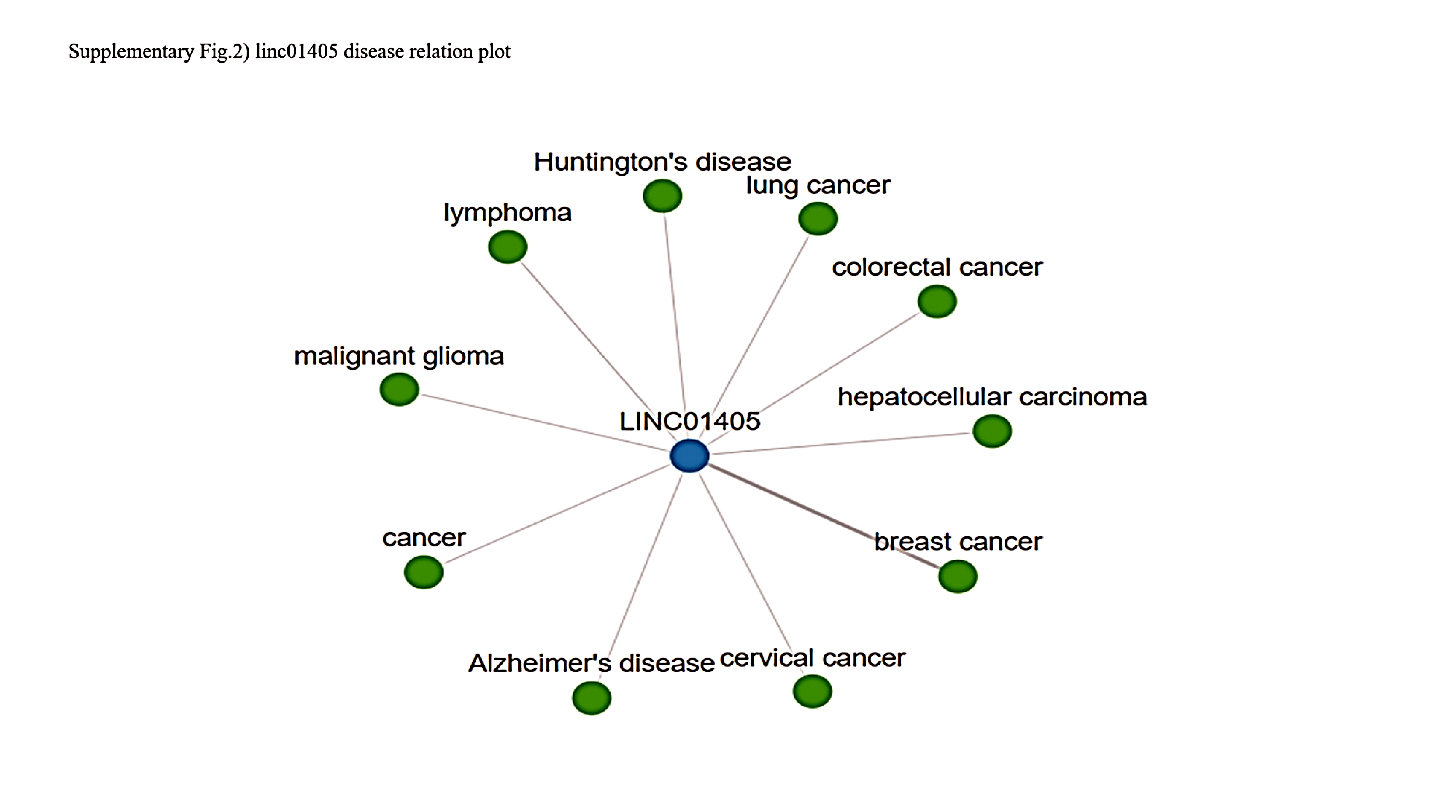
**

**Supplementary Fig.3) linc01405 disease relation plot: linc01405 disease assessment based on LncRNADisease v2.0 (http://www.rnanut.net/lncrnadisease/index.php/hom**

**
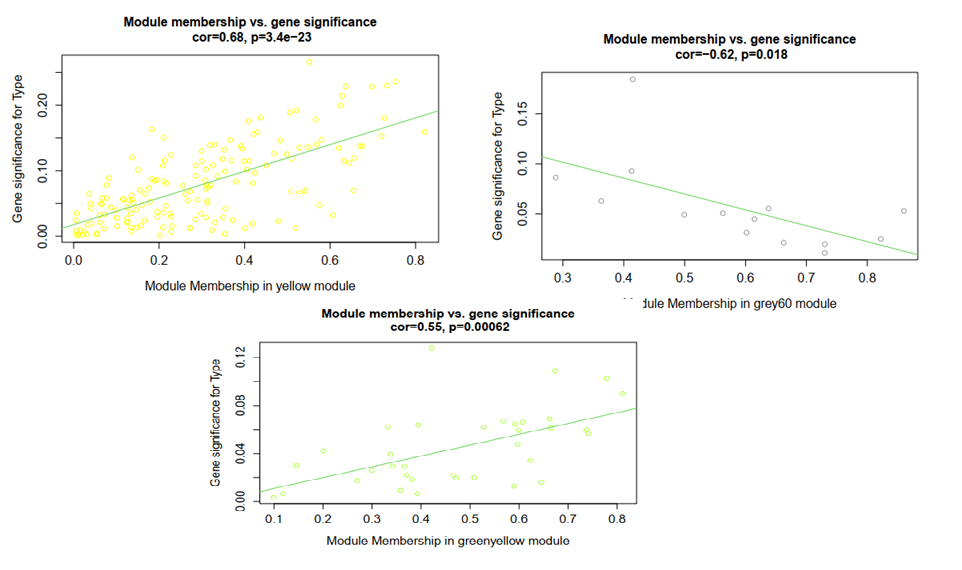
**

**Supplementary Fig.4) Scatter plot of the key modules (yellow, gray60, green-yellow) representing breast cancer stages.**


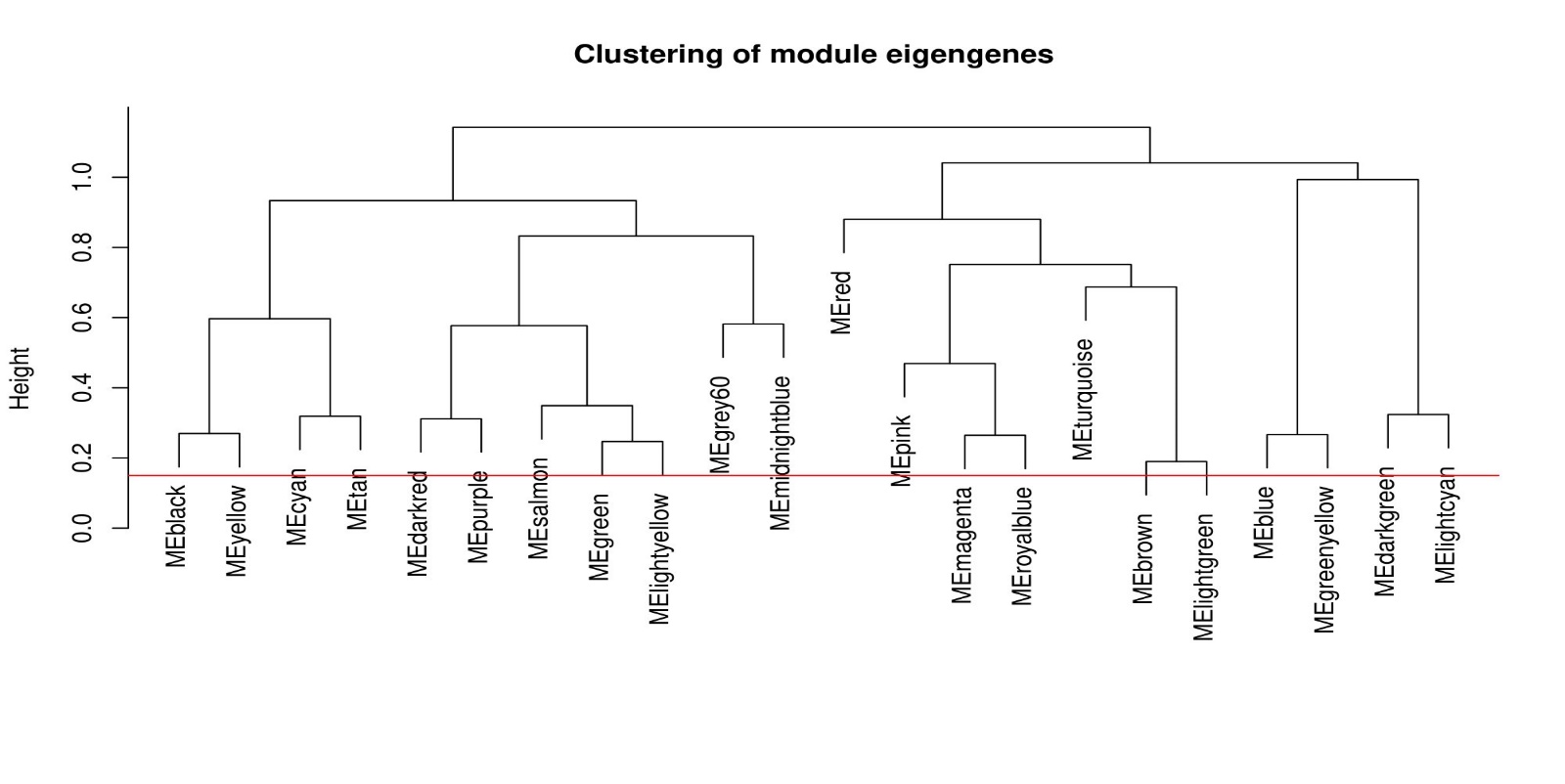
**Supplementary Fig.5) Shows the clustering of module eigengenes, in which 22 modules are produced. Only two modules (MEbrown and MElightgreen) are lower than the cutoff (red line).**


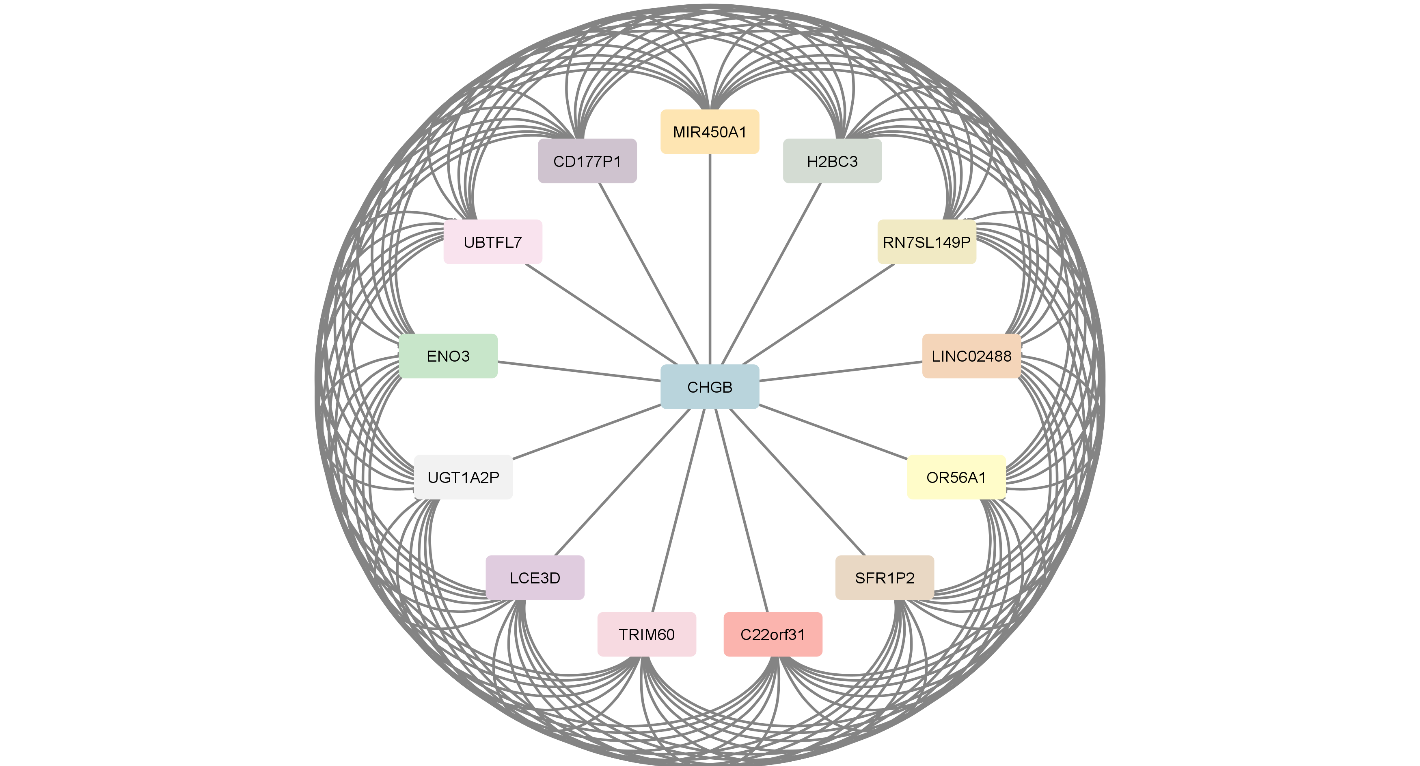


**Supplementary Fig.6) Representation of** **gray modules produced by WGCNA analysis in breast cancer**.


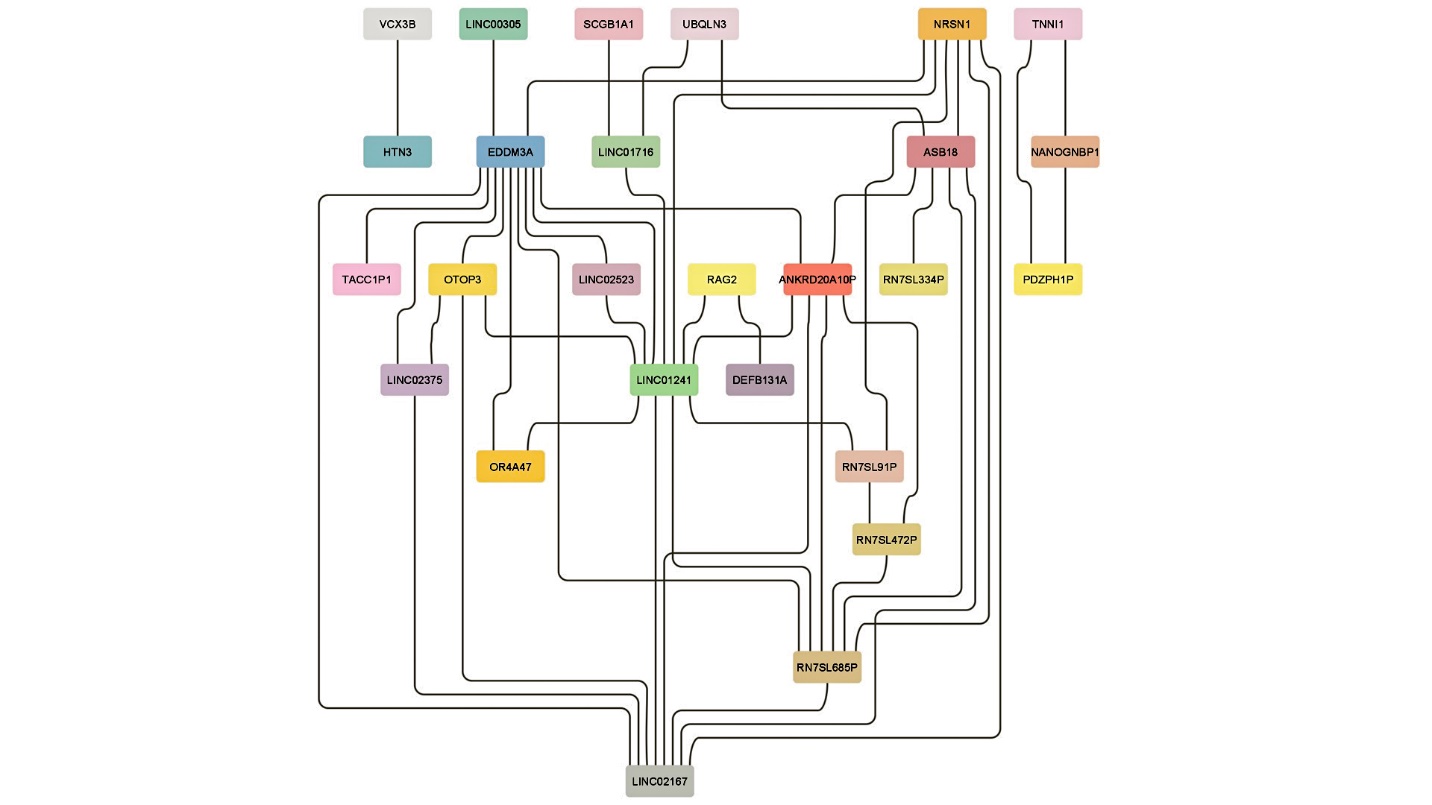


**Supplementary Fig.7) Representation of** **yellow** **modules produced by WGCNA analysis in breast cancer**.


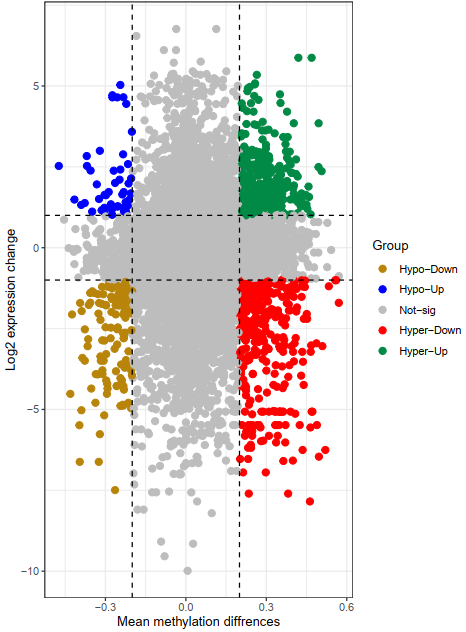

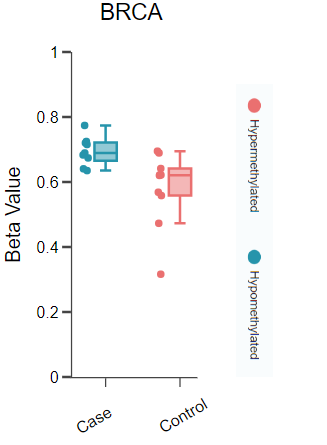


**Supplementary Fig.8)**

A) Scatter plot of mean methylation difference versus log2 expression

B) Methylation status of Linc01405

**
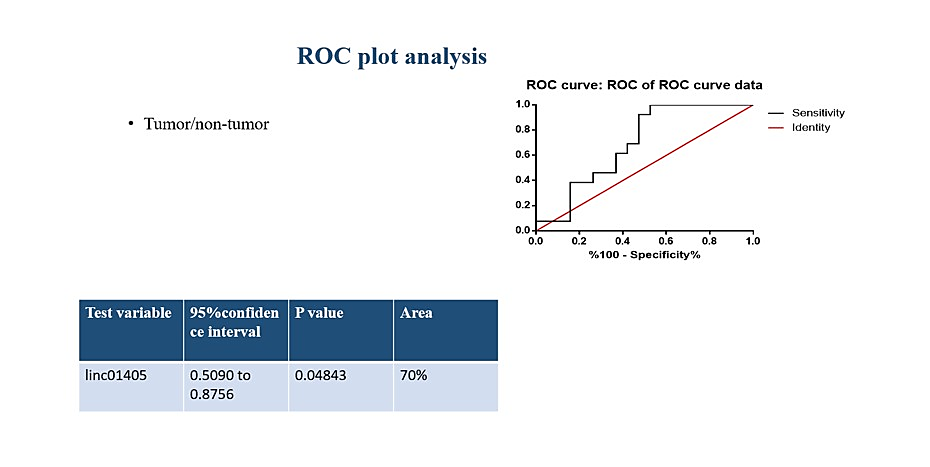
**

**Supplementary Fig.9) ROC curve analysis of Linc01405 based on Real-Time data, with the area under the curve at 70 percent.**


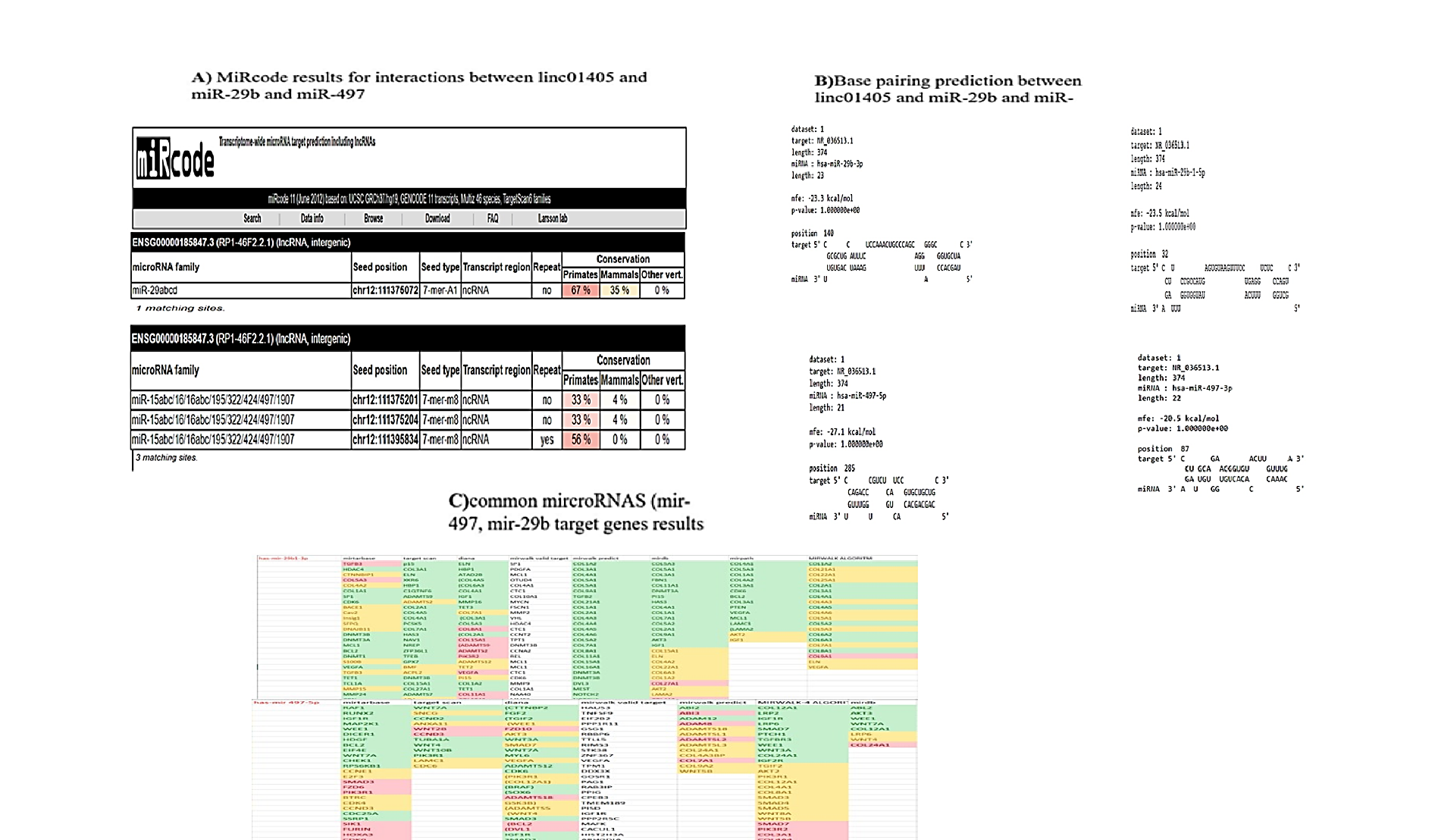


**Supplementary Fig.10) Bioinformatics analysis of microRNAS sponges and targets**

A. MiRcode results from interactions between linc01405 and miR-29b and miR-497

B. Base pairing prediction between linc01405 and miR-29b and miR-497

Common mircroRNAS (mir-497, mir-29b target genes result
