# Supplementary material for: In‐silico and in‐vitro evidence suggest LINC01405 as a sponge for miR‐29b and miR‐497‐5p, and a potential regulator of Wnt, PI3K, and TGFB signaling pathways in breast carcinoma
Source: Cancer Rep (Hoboken). 2024 Jan 15;7(2):e1972. doi: 10.1002/cnr2.1972 (PMC10849987; doi:10.1002/cnr2.1972)
Supplement: Supplementary file 2 — Supplementary Table 1. The top 20 most strongly up‐ or down‐regulated DEGs by meta‐analysis of microarray data. Supplementary Table 2. The top 20 most strongly up‐ or downregulated DEGs in a meta‐analysis of microarray data and GSE68086. Supplementary Table 3. The top 20 most strongly up‐ or down‐regulated DEGs in TCGA Supplementary Table 4. list of primers. Supplementary Table 5. differentially expressed and methylated genes. [file CNR2-7-e1972-s001.docx]

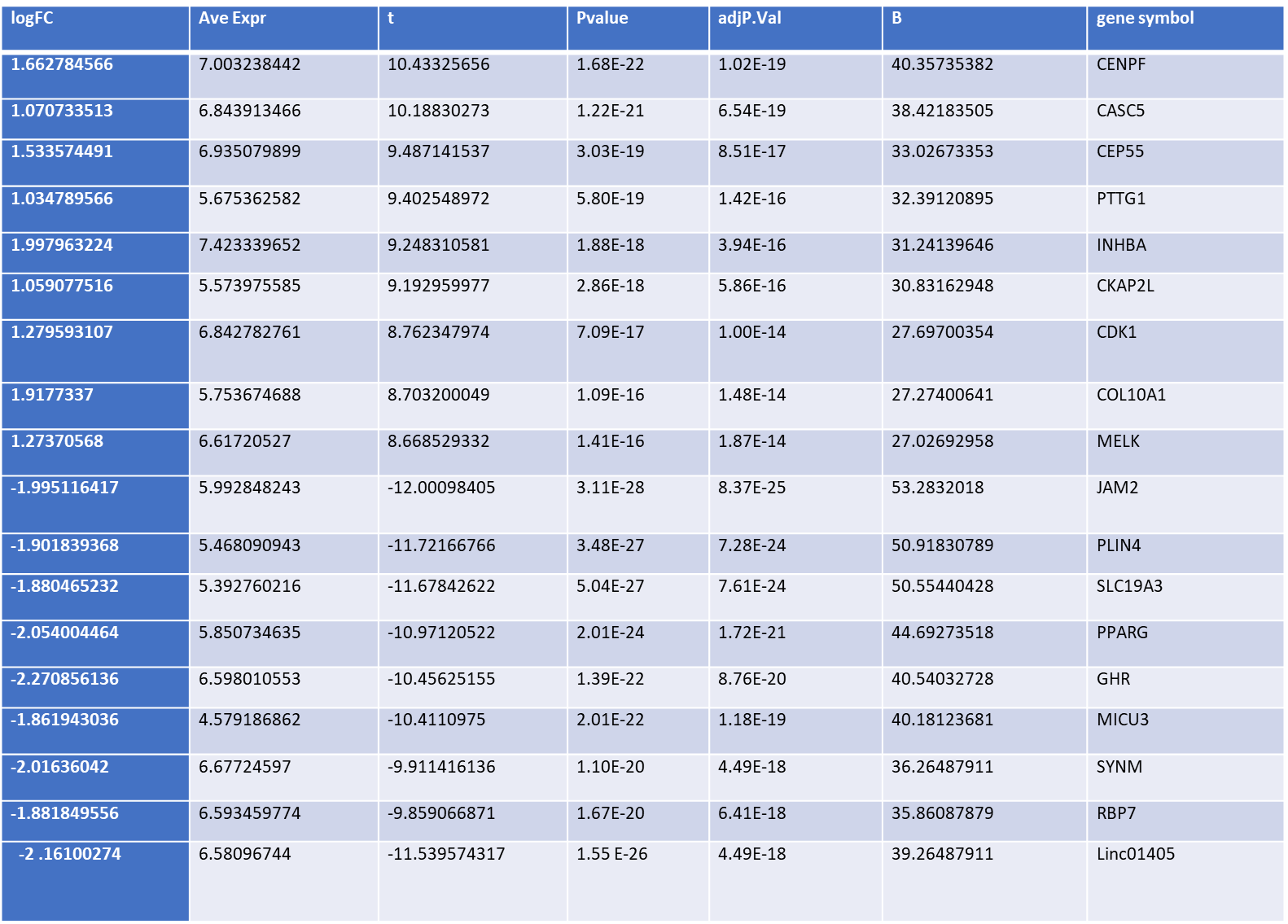


**Supplementary Table 1) The top 20 most strongly up- or down-regulated DEGs by meta-analysis of microarray data.**


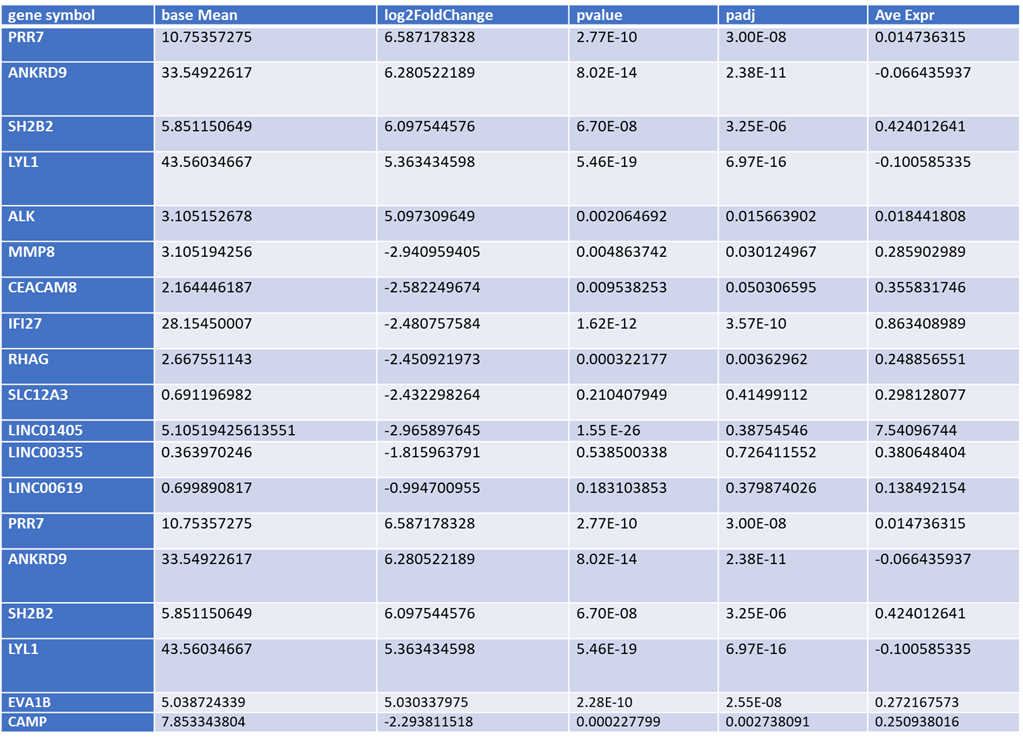


**Supplementary Table 2) The top 20 most strongly up- or downregulated DEGs in a meta-analysis of microarray data and GSE68086.**


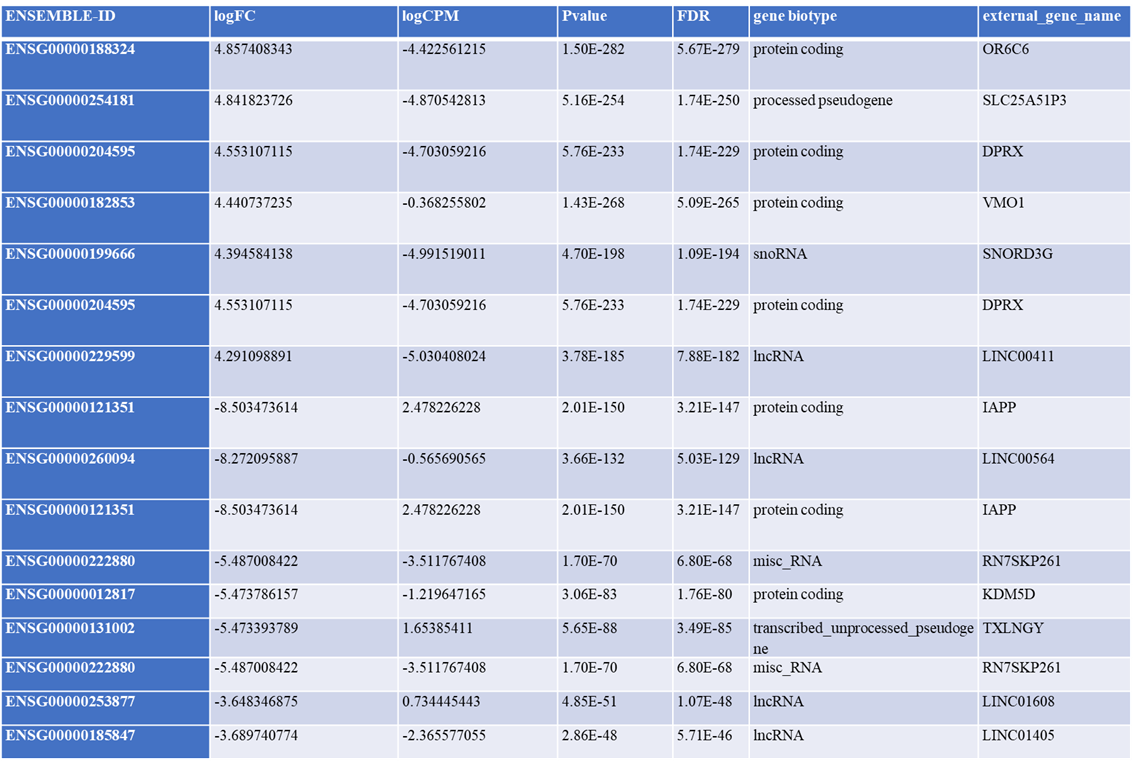
**Supplementary Table 3) The top 20 most strongly up- or down-regulated DEGs in TCGA.**


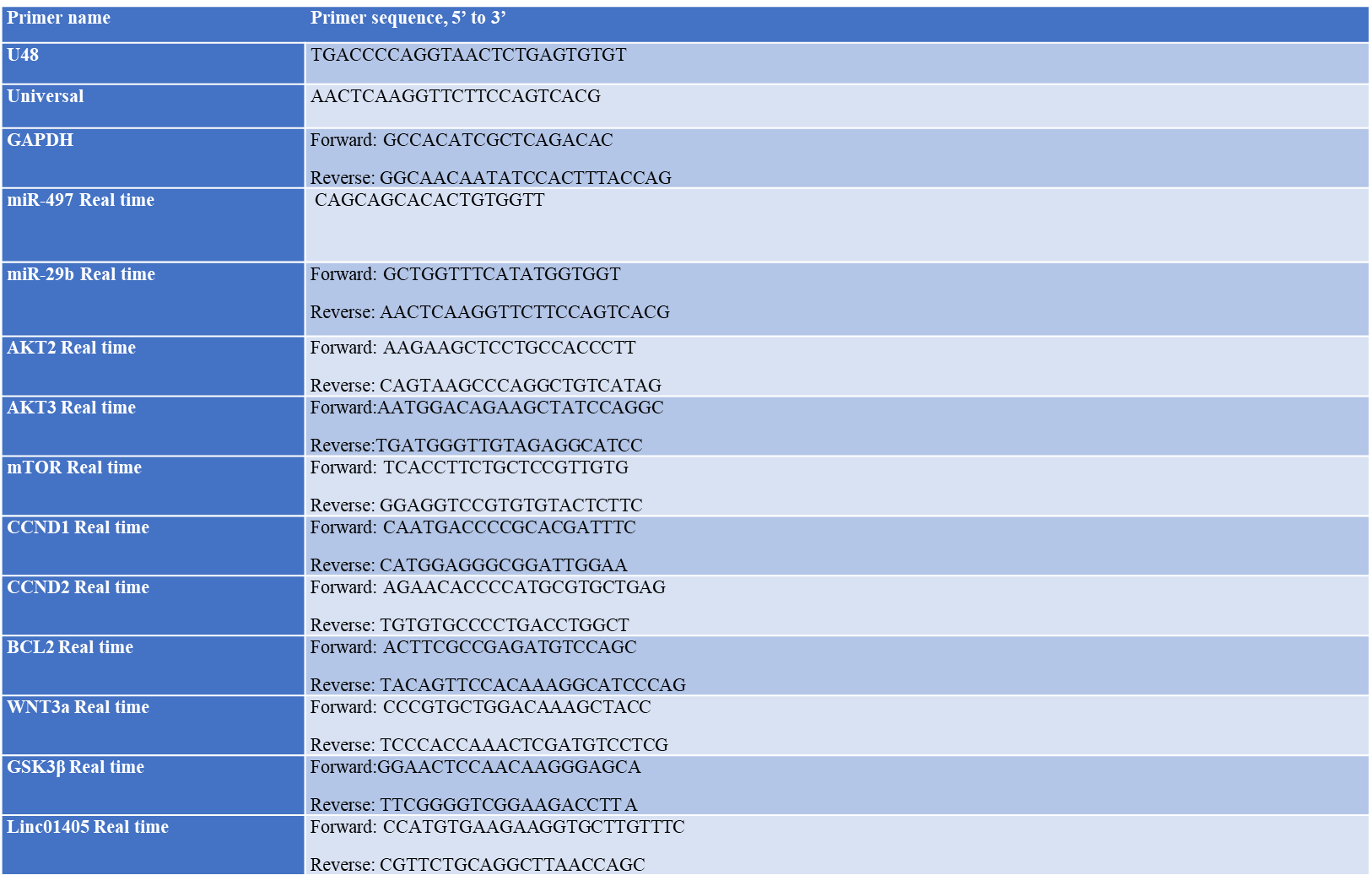


**Supplementary Table 4) list of primers.**

| external_gene_name | logFC | FDR | gene biotype | delta beta | adj pvalue |
| --- | --- | --- | --- | --- | --- |
| A1BG | -1.246600399 | 6.05E-18 | protein coding | 0.006892277 | 8.15E-08 |
| A1BG-AS1 | -0.709318527 | 2.77E-07 | lncRNA | 0.006892277 | 8.15E-08 |
| A2M | 0.89456188 | 1.55E-14 | protein coding | 0.268096553 | 3.65E-09 |
| A2ML1 | -3.789033408 | 4.18E-107 | protein coding | -0.0484244 | 0.590354452 |
| A2ML1 | -3.789033408 | 4.18E-107 | protein coding | -0.04778687 | 0.593962622 |
| A4GALT | 0.307493771 | 0.015266841 | protein coding | -0.025964807 | 0.822889779 |
| A4GALT | 0.307493771 | 0.015266841 | protein coding | -0.025550249 | 0.00012217 |
| AAAS | -0.245748273 | 0.075212116 | protein coding | -0.048044685 | 1.06E-07 |
| AAAS | -0.245748273 | 0.075212116 | protein coding | 0.025220448 | 0.000104052 |
| AACS | 0.510102652 | 3.07E-05 | protein coding | -0.011975369 | 4.42E-05 |
| AADAC | 2.868008715 | 1.50E-189 | protein coding | -0.04027655 | 0.101772639 |
| AADAT | -0.429205734 | 0.001724004 | protein coding | 0.037747523 | 0.362396254 |
| AADAT | -0.429205734 | 0.001724004 | protein coding | 0.075848523 | 6.73E-06 |
| AAGAB | -0.841681672 | 1.28E-09 | protein coding | 0.001182704 | 0.405430003 |
| AAK1 | 0.051748272 | 0.702079278 | protein coding | 0.00313644 | 0.082169832 |
| AAMDC | -0.0030177 | 0.999466727 | protein coding | -0.041601844 | 0.115965211 |

**Supplementary Table 5) differentially expressed and methylated genes.**
